# Microparticle alpha-2-macroglobulin enhances pro-resolving responses and promotes survival in sepsis

**DOI:** 10.1002/emmm.201303503

**Published:** 2013-12-16

**Authors:** Jesmond Dalli, Lucy V Norling, Trinidad Montero-Melendez, Donata Federici Canova, Hazem Lashin, Anton M Pavlov, Gleb B Sukhorukov, Charles J Hinds, Mauro Perretti

**Affiliations:** 1 Centre for Biochemical Pharmacology, The William Harvey Research Institute, Barts and The London School of Medicine, Queen Mary University of LondonLondon, UK; 2 School of Engineering & Materials Science, Queen Mary University of LondonLondon, UK; 3 Intensive Care Unit, St. Bartholomew's Hospital, Barts HealthLondon, UK; † Center for Experimental Therapeutics and Reperfusion Injury, Harvard Institute of Medicine, Brigham and Women's Hospital and Harvard Medical SchoolBoston, MA, USA

**Keywords:** activation, innate immunity, lipoprotein receptor-related protein 1, neutrophil, phagocytosis

## Abstract

Incorporation of locally produced signaling molecules into cell-derived vesicles may serve as an endogenous mediator delivery system. We recently reported that levels alpha-2-macroglobulin (A2MG)-containing microparticles are elevated in plasma from patients with sepsis. Herein, we investigated the immunomodulatory actions of A2MG containing microparticles during sepsis. Administration of A2MG-enriched (A2MG-E)-microparticles to mice with microbial sepsis protected against hypothermia, reduced bacterial titers, elevated immunoresolvent lipid mediator levels in inflammatory exudates and reduced systemic inflammation. A2MG-E microparticles also enhanced survival in murine sepsis, an action lost in mice transfected with siRNA for LRP1, a putative A2MG receptor. *In vitro*, A2MG was functionally transferred onto endothelial cell plasma membranes from microparticles, augmenting neutrophil–endothelial adhesion. A2MG also modulated human leukocyte responses: enhanced bacterial phagocytosis, reactive oxygen species production, cathelicidin release, prevented endotoxin induced CXCR2 downregulation and preserved neutrophil chemotaxis in the presence of LPS. A significant association was also found between elevated plasma levels of A2MG-containing microparticles and survival in human sepsis patients. Taken together, these results identify A2MG enrichment in microparticles as an important host protective mechanism in sepsis.

## Introduction

A coordinated host response is a pre-requisite for successful protection against injury and infection. It is now appreciated that both the initiation (Gilroy *et al*, 2004) and resolution (Spite & Serhan, 2010; Levy *et al*, 2012; Ortega-Gomez *et al*, 2013) of inflammation are active processes that under optimal conditions are tightly regulated to achieve clearance of infection and repair of damaged tissues, favoring return to homeostasis. Recent work employing quantitative methods to assess cellular trafficking at the site of injury and/or infection and the production of mediators during the course of inflammation-resolution has shed light on some of the mechanisms involved in self-limited versus delayed resolution (Bannenberg *et al*, 2005; Stables *et al*, 2011; Chiang *et al*, 2012). These studies demonstrate that leukocyte recruitment and trafficking at the site of inflammation is regulated via the production of mediators that signal the onset as well as termination of the inflammatory response. Failure of the host to engage these pathways results in an aberrant response and persistent inflammation (Stables *et al*, 2011; Chiang *et al*, 2012; Barnig *et al*, 2013).

Inability of the host to contain an invading pathogen is associated with a systemic inflammatory response (sepsis) that, when dysregulated, can precipitate impaired tissue perfusion leading to organ injury (severe sepsis) with or without hypotension (septic shock). Lack of progress in combating the high mortality and morbidity associated with severe sepsis, in part reflects our limited understanding of the complex biological pathways that regulate innate immunity (Riedemann *et al*, 2003; Goldenberg *et al*, 2011).

A novel mode of non-soluble communication between cells has recently emerged centered around plasma membrane-shed vesicles, termed microparticles (Distler & Distler, 2010), which complement intercellular communication driven by soluble mediators (e.g. cytokines and autacoids). The incorporation of mediators, including proteins and lipid mediators, into vesicles is an efficient means of long range cellular communication since microparticles facilitate trafficking of these mediators from the site of origin minimizing dilution and/or inactivation (Kunder *et al*, 2009; Norling & Dalli, 2013).

Since alpha-2-macroglobulin (A2MG) levels are elevated in plasma neutrophil microparticles from a small cohort of septic patients when compared to plasma microparticles from healthy volunteers (Dalli *et al*, 2013), we investigated the actions of A2MG containing microparticles in regulating the host responses during sepsis. Administration of A2MG enriched (A2MG-E) microparticles following caecal ligation and puncture (CLP) in mice activated the host endogenous pro-resolving pathways and significantly reduced mortality. A2MG was also found to preserve human leukocyte responses where it promoted neutrophil chemotaxis and adhesion in the presence of endotoxin as well as stimulated bacterial phagocytosis by leukocytes.

## Results

### A2MG enriched microparticles are protective in murine sepsis and increase exudate pro-resolving lipid mediator levels

**Figure 1:**
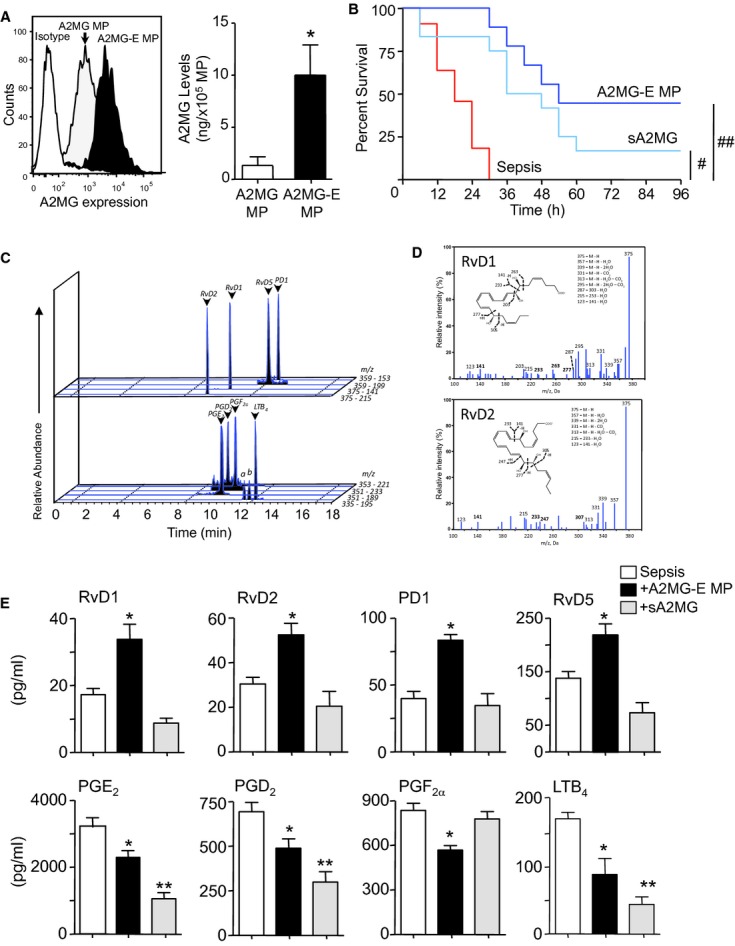
A2MG-E microparticles enhance survival in murine sepsis and elevate exudate pro-resolving lipid mediator levels. A  A2MG containing microparticles (A2MG MP) were further enriched with A2MG (A2MG-E MP) and incorporation assessed by flow cytometry and ELISA. B  Kaplan–Meier survival analysis of mice subjected to CLP at time 0 (see Materials and Methods for details) and 1 h later treated with PBS (100 μl i.v.), soluble A2MG (sA2MG; 0.05 μg/mouse i.v.) or A2MG-E MP (1 × 10^5^/mouse i.v.). Results are mean ± s.e.m., *n* = 12 mice per group (^#^*P *< 0.01, ^##^*P *< 0.001 versus PBS treated mice, one tailed log rank test). C–E  CLP mice were treated as in B and sacrificed at 12 h. Lipid mediators in peritoneal exudates were assessed using lipid mediator metabololipidomics following solid phase extraction (see Materials and Methods for details). C  Representative multiple reaction monitoring chromatograms of the identified lipid mediators in the peritoneal exudates. D  Accompanying MS-MS spectra employed for identification. E  Quantification of the identified lipid mediators was achieved by multiple reaction monitoring using parent ion and diagnostic ion in the MS-MS (daughter ion). Results for E are mean ± s.e.m., *n* = 4–5 mice per group (*P < 0.05 versus PBS treated mice by one way ANOVA).

To determine the role of A2MG containing microparticles in sepsis we utilized human neutrophil microparticles containing A2MG (Dalli *et al*, 2013). We further enriched these microparticles with A2MG employing an established method that uses energy-induced conversion (see Materials and Methods for details) and allows for the incorporation of target products into membrane bound vesicles (Norling *et al*, 2011). Using this protocol we obtained microparticles with ∼8 times higher A2MG content (1.3 ± 0.9 versus 10 ± 2.2 ng/1 × 10^5^ microparticles; termed A2MG-E microparticles; Fig 1A and supplementary Fig S1). We next treated mice with PBS, A2MG-E microparticles (1 × 10^5^/mouse) or the free protein (i.e. not incorporated into microparticles; sA2MG), 1 h post CLP, a well-established model of microbial sepsis (Rittirsch *et al*, 2009). Here we employed similar amounts of sA2MG to those found in the A2M-E microparticles in order to investigate whether protective actions of A2MG were dependent on incorporation into microparticles. Administration of A2M-E microparticles led to a significant reduction in 4-day mortality when compared to mice that were administered PBS or sA2MG (Fig 1B). sA2MG also afforded a lesser degree of protection from death when compared to PBS treated mice (Fig 1B).

Having observed that A2M-E microparticles were protective in murine sepsis we next investigated whether they modulated the humoral response by analyzing 12 h peritoneal exudates. Using lipid mediator metabololipidomics we identified high levels of mediators from both the lipoxygenase and cyclooxygenase pathways including prostaglandin (PG) D_2_, leukotriene (LT) B_4_ and resolvin (Rv) D2 (Fig 1C). These mediators were identified in accordance with published criteria (Yang *et al*, 2011) that included matching retention times, fragmentation patterns and at least 6 characteristic and diagnostic ions for each as illustrated for RvD1 and RvD2 (Fig 1D). Lipid mediator quantification was achieved by multiple reaction monitoring of signature ion pairs for the parent ion (Q1) and a characteristic daughter ion (Q3; Fig 1C). Administration of both A2MG-E microparticles and sA2MG significantly reduced pro-inflammatory lipid mediator levels including PGD_2_ (∼20–40%, *P *< 0.05; Fig 1E) and the potent chemoattractant LTB_4_ (>50%; *P *< 0.05). Of note, administration of A2MG-E microparticles, but not sA2MG, increased exudate pro-resolving lipid mediator levels including RvD1, RvD5 and the potent immunoresolvent RvD2 (∼100% increase, *P *< 0.05; Fig 1E). Collectively these findings demonstrate that A2MG-E microparticles promote a pro-resolving lipid mediator profile in inflammatory exudates from CLP mice (supplementary Fig S2A and B), an action that was not shared with sA2MG.

### Microparticles enriched in A2MG enhance bacterial clearance and reduced inflammation in murine sepsis

**Figure 2:**
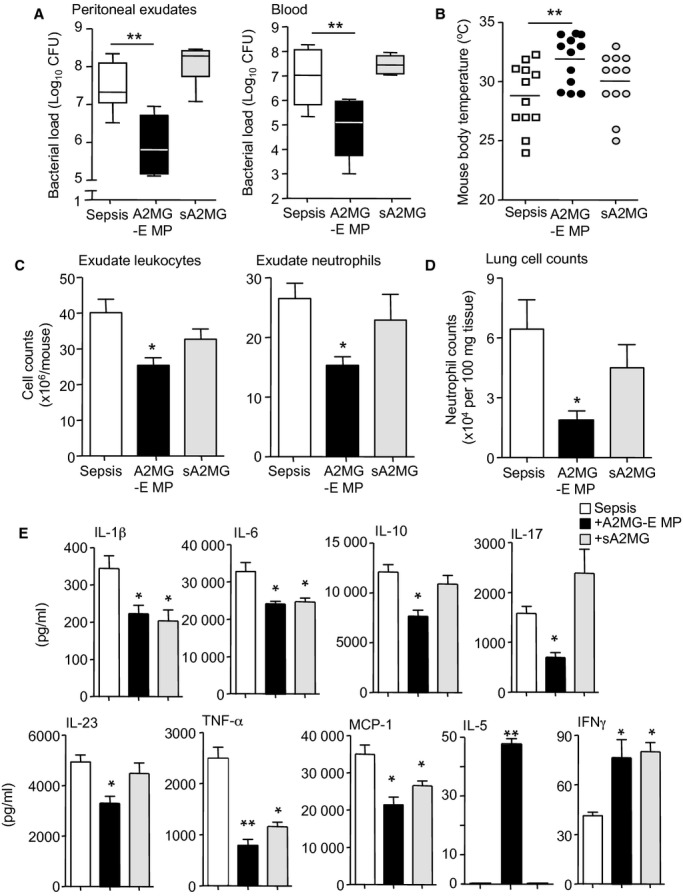
Bacterial containment and clearance is enhanced by A2MG-E microparticles reducing local and systemic inflammation during microbial sepsis. Mice were subjected to CLP (see Materials and Methods for details) 1 h later treated with PBS (100 μl i.v.), A2MG-E MP (1 × 10^5^ i.v.) or sA2MG (0.05 μg) and sacrificed at 12 h. Results A, C–E are mean ± s.e.m. A, C–E, *n* = 6 mice per group; B, *n* = 12 mice per group (*P < 0.05, **P < 0.01 versus PBS treated mice by one way ANOVA). A  Aerobic bacterial levels in peritoneal exudates and blood from CLP mice. B  Body temperature at 12 h following CLP. C  Peritoneal exudate leukocyte and neutrophil counts. D  Counts for trapped neutrophils in lungs, as determined by light microscopy, Ly6G staining and flow cytometry. E  Exudate cytokine levels as determined by multiplex array.

Since bacterial containment and clearance are critical to controlling inflammatory responses during sepsis we next investigated whether microparticle incorporated A2MG also regulated these protective processes. Administration of A2MG-E microparticles 1h post CLP, markedly reduced local and systemic bacterial loads (Fig 2A) and protected against hypothermia (Fig 2B). Administration of sA2MG did not lead to a significant reduction in bacterial loads or protection from hypothermia. Of note, A2MG did not display direct anti-bacterial actions (supplementary Fig S3), thereby suggesting that the enhanced bacterial containment and clearance found *in vivo* after A2MG-E microparticles administration resulted from direct modulation of the host responses.

Assessment of exudate leukocyte counts demonstrated a reduction in local inflammation 12 h post-CLP in mice treated with A2MG-E microparticles as evidenced by lower exudate leukocyte levels (∼40% reduction in neutrophil counts; *P *< 0.05; Fig 2C). Of note this reduction in leukocytes was primarily due to a significant reduction in neutrophils but not monocytes/macrophages (supplementary Fig S4A) leading to a significant increase in the monocyte/macrophage to neutrophil ratio (supplementary Fig S4B), a hallmark of resolution (Spite *et al*, 2009; Chiang *et al*, 2012). Assessment of exudate cytokine levels demonstrated a significant decrease in pro-inflammatory cytokines including IL-1β (∼80%, *P *< 0.05) and TNF-α (∼50%, *P *< 0.05; supplementary Fig S2C) in A2MG-E microparticles treated animals. A2MG-E microparticles administration also led to a decrease in systemic inflammation as demonstrated by: (i) a reduction in neutrophil infiltration into the lungs (∼65% reduction *P *< 0.05; Fig 2D) and (ii) lower plasma pro-inflammatory cytokine levels, including IL-6 (∼35%, *P *< 0.05) and MCP-1 (∼70%, *P *< 0.05; Fig 2E). Assessment of circulating white blood cell counts demonstrated an increase in circulating leukocyte levels following A2MG-E microparticle administration (supplementary Fig S4C). This is in keeping with a reduction in secondary organ injury and thus a reduction in neutrophil trafficking to non-infected organs, a hallmark of sepsis (Qiu *et al*, 2000). A2MG-E microparticles did not reduce the soluble response *tout-court*, since we also found a specific and significant elevation in local and circulating levels of IFN-γ (∼100% increase; *P *< 0.05) and IL-5 (Fig 2E and supplementary Fig S2C).

Administration of sA2MG following CLP also led to a reduction in pro-inflammatory cytokine levels in murine exudates and plasma that included MCP-1 and IL-1β (Fig 2E and supplementary Fig S2C), yet this treatment did not reduce exudate leukocyte levels (Fig 2C) nor did it protect against second organ injury (Fig 2B and D). We also observed a significant reduction in monocyte/macrophage levels in the CLP exudates when compared to both PBS and A2MG-E microparticles treated mice (supplementary Fig S4A). In addition there were no differences in the number of circulating leukocytes when compared to PBS treated mice (supplementary Fig S4C). These findings suggest that A2MG incorporation into microparticles markedly enhances the protective action of this protein in murine sepsis, resulting in selective modulation of cytokines and immune-modulatory lipid mediators as well as enhanced bacterial killing that lead to a reduction in local and systemic inflammation.

### A2MG microvesicles display protective actions in murine sepsis

**Figure 3:**
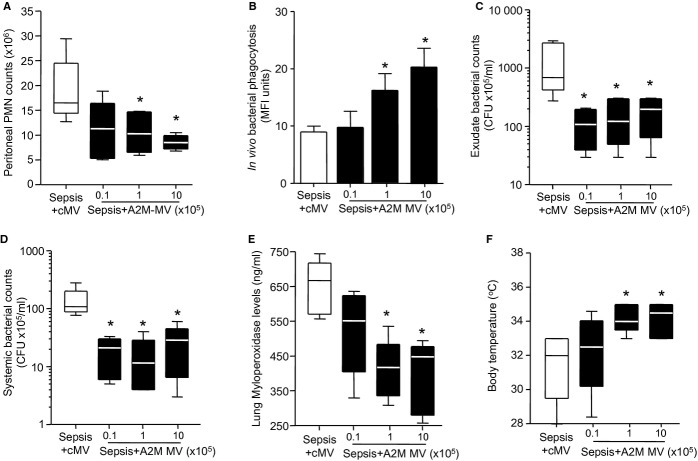
A2MG microvesicles dose dependently stimulate bacterial clearance in vivo reducing local and systemic inflammation during microbial sepsis. Microvesicles (cMV; 10^6^ capsules per mouse) or microvesicles containing A2MG (A2MG-MV) were administered i.v. at the indicated doses 5 min prior to CLP (see Materials and Methods for details). Mice were sacrificed at 12 h. Results are mean ± s.e.m. *n* = 6 mice per group (*P < 0.05, **P < 0.01 versus cMV treated mice by one way ANOVA). A  Peritoneal exudate leukocyte counts. B  Exudate bacterial phagocytosis determined by flow cytometry. C,D  Aerobic bacterial levels in (C) peritoneal exudates and (D) blood from CLP mice. E  Lung MPO levels. F  Body temperature at 12 h following CLP.

**Figure 4:**
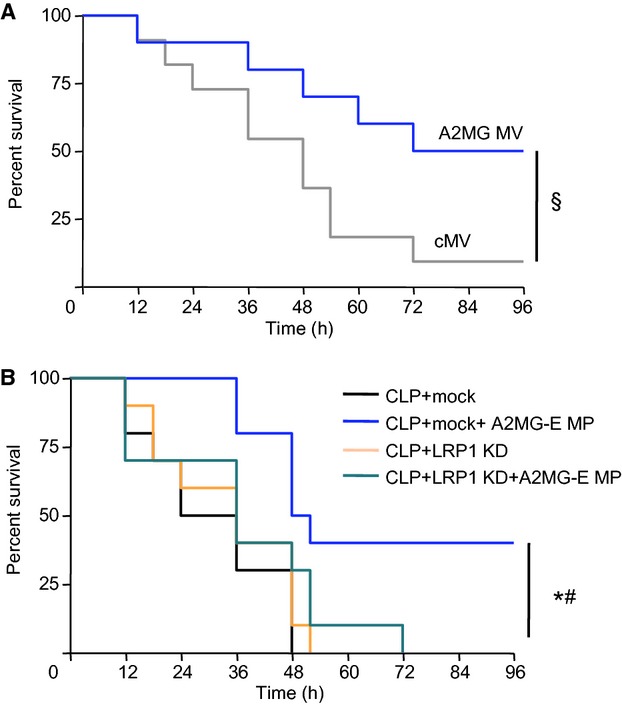
LRP1 mediates the protective actions of A2MG during microbial sepsis. Results are mean ± s.e.m., *n* = 10 mice per group (^§^*P* < 0.05 versus cMV treated mice; *P < 0.05, versus PBS mice; ^#^*P* < 0.05, versus LRP1 KD A2M-E MP mice by one tailed log rank test). A  Microvesicles (cMV; 10^6^/mouse) or microvesicles containing A2MG (A2MG-MV 10^6^/mouse) were administered i.v. 5-min prior to CLP (see Materials and Methods for details) and survival assessed over a 96 h interval. B  Mice were transfected with either mock or LRP1 siRNA (LRP1 KD see Materials and Methods for details). After 48 h PBS or A2MG-E MP (1 × 10^5^ i.v.) were administered i.v. 5 min prior to CLP.

Since A2MG microparticles contain a number of proteins (Dalli *et al*, 2013) that may also exert protective actions we next constructed synthetic microvesicles that were selectively loaded with a defined amount of A2MG (A2MG-MV; see Materials and Methods for details) and tested them in murine sepsis. Administration of A2MG-MV to CLP mice led to a dose dependent reduction in peritoneal exudate neutrophil counts at 12 h when compared with mice treated with control microvesicles (cMV; Fig 3A). Treatment of mice with A2MG-MV also led to a dose-dependent increase in the number of phagocytosed bacteria in murine peritoneal exudates, as determined by *in situ* hybridization and flow cytometry (Fig 3B). Furthermore, there was a dose dependent reduction in both exudate (Fig 3C) and plasma (Fig 3D) bacterial loads when compared to mice treated with cMV. Administration of A2MG-MV also led to a dose dependent reduction in exudate and plasma levels of pro-inflammatory cytokines, including IL6 and TNF-α (supplementary Fig S5) as well as second organ injury, as determined by a reduction in lung MPO levels (Fig 3E). Treatment of mice subjected to CLP with A2MG-MV significantly protected against hypothermia (Fig 3F). In all cases, doses of 1 × 10^5^ and 1 × 10^6^ A2MG-MV elicited the most significant effects. Administration of A2MG-MV (1 × 10^6^) led to a significant reduction in mortality when compared with mice treated with cMV (Fig 4A). Together these results indicate that A2MG exerts protective actions in sepsis likely mediating at least some of the protective actions displayed by A2MG-E MP (Figs 1 and 2).

To corroborate this further, we next investigated whether the A2MG receptor, the low-density lipoprotein receptor-related protein (LRP) 1, mediated these protective actions. Here we employed an established *in vivo* transfection system using siRNA to silence LRP1 expression in mice. Mice received either a mock lentiviral vector or a lentiviral vector containing a siRNA sequence to LRP1. Assessment of peripheral blood LRP1 levels 48 h after transfection demonstrated a significant reduction in peripheral blood LRP1 expression (∼50%; *P* < 0.05; supplementary Fig S6), confirming the efficacy of this approach at reducing LRP1 expression in peripheral blood leukocytes. We next subjected mock transfected or siRNA transfected mice to CLP, then treated these mice with either PBS or A2M-E MP and assessed survival over 4 days. There were no significant differences between the two vehicle treated groups with 100% lethality within 52 h (Fig 4). Administration of A2M-E MP to mice treated with mock vector significantly improved survival, an action that was lost in mice that were also treated with LRP1 siRNA (Fig 4). Together these results indicate that in mice LRP1 mediates the protective actions of A2MG-E MP.

### LRP1 mediates the actions of A2MG on human leukocytes

**Figure 5:**
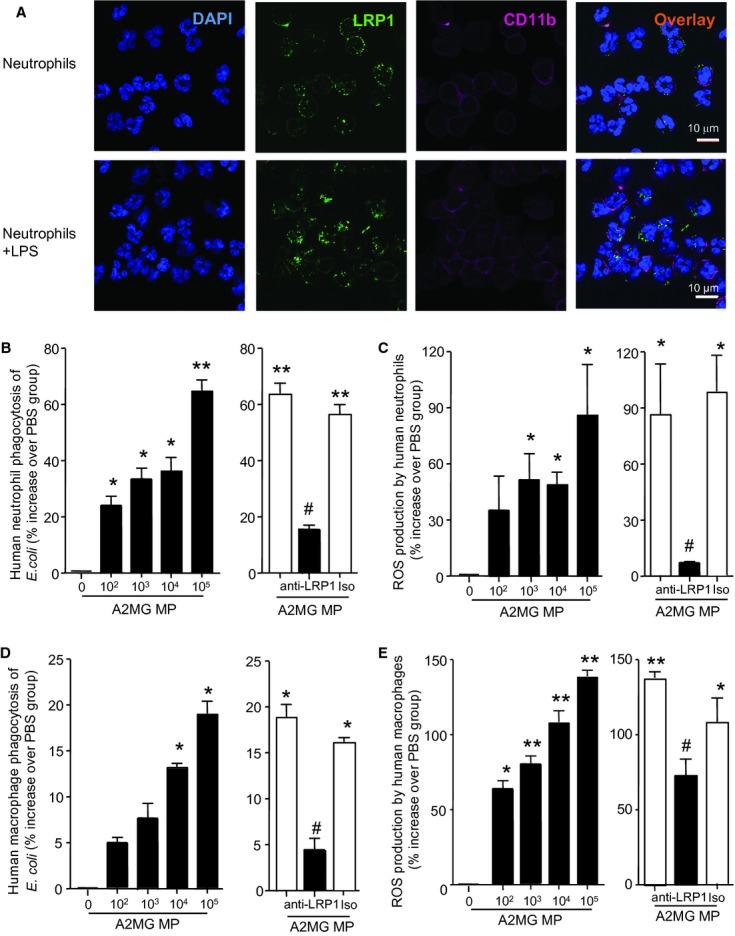
A2MG stimulates bacterial phagocytosis by human leukocytes via LRP1. Results are mean ± s.e.m. of three individual cell preparations (*P < 0.05, **P < 0.01 versus PBS incubation; ^#^*P* < 0.05 versus A2MG MP incubation by one way ANOVA). A  LRP1 expression was monitored on resting and LPS-stimulated human neutrophils (1 μg/ml, 1 h, 37°C) by confocal microscopy (see Materials and Methods for details). Results are representative of *n* = 3 individual cell preparations. B–E  Human neutrophil and macrophage bacterial phagocytosis and ROS production. B  Peripheral blood human neutrophils (1 × 10^5^/well). C  Primary human macrophages (1 × 10^4^/well) were plated in 96 well plates and incubated with PBS or A2MG MP (10^2^–10^5^ microparticles per well) 15 min prior to the addition of BacLight labeled *E. coli* (1:50 neutrophil/macrophage to bacteria) for 60 min (37°C). In some instances anti-LRP1 (1 μg/ml) antibody was added 30 min prior to the addition of A2MG (right panel). D  Peripheral blood human neutrophils (1 × 10^5^/well). E  Primary human macrophages (1 × 10^4^/well) were incubated CM-H_2_DCFDA for 30 min prior to plating in 96 well plates. These were then incubated with PBS or A2MG MP (10^2^–10^5^ microparticles per well) 15 min prior to the addition of *E. coli* (1:50 neutrophil/macrophage to bacteria) for 30 min (37°C) and intracellular ROS production was determined on a plate reader. In some instances anti-LRP1 (1 μg/ml) antibody was added 30 min prior to the addition of A2MG MP (right panel).

Since A2MG containing microparticles demonstrated protective actions in murine sepsis we next investigated the cellular and molecular mechanisms activated by microparticle-A2MG using isolated human and mouse leukocytes. We used flow cytometry to assess the relative distribution of LRP1 on human neutrophils. Here we found that LRP1 immunoreactivity was higher in permeabilized cells in comparison to non-permeabilized cells (supplementary Fig S7A) demonstrating that this protein was expressed both on the plasma membrane and, at higher levels, in the cytosol. Confocal analysis corroborated LRP1 expression on the plasma membrane of resting human neutrophils (Fig 5A). Assessment of LPR1 expression following exposure to LPS demonstrated a significant upregulation of LRP1 expression on human neutrophils plasma membrane (Fig 5A and supplementary Fig S7B). Incubation of mouse neutrophils with LPS also led to a significant increase in cell membrane LRP1 expression (supplementary Fig S7C). These results suggest that neutrophil activation leads to LRP1 mobilization from intracellular stores to the plasma membrane.

Next we investigated the role of LRP1 in mediating the protective responses of microparticle-A2MG on human primary leukocytes. Incubation of neutrophils with microparticles containing elevated A2MG levels, [termed A2MG MP; see Materials and Methods and Dalli *et al* (2013) for details] led to a dose dependent increase in bacterial phagocytosis (Fig 5B), an action that was inhibited when leukocytes were pre-incubated with an anti-LRP1 antibody. When human neutrophils were incubated with A2MG MP we found a significant increase in ROS production when the neutrophils were also incubated with *Escherichia coli* (Fig 5C), an action inhibited when cells were pre-incubated with an anti-LRP1 antibody. Similar results were obtained with macrophages both in terms of bacterial phagocytosis (Fig 5D) and ROS production (Fig 5E). These results indicate that leukocyte LRP1 mediates the protective actions of A2M MP.

In order to assess the role of A2MG in regulating the leukocyte responses elicited by A2MG MP we next investigated whether sA2MG regulated macrophage and peripheral blood neutrophil phagocytosis and ROS production. Incubation of human and mouse macrophages with sA2MG led to a dose dependent increase in macrophage phagocytosis (supplementary Figs S8 and S9). Incubation of human macrophages with sA2MG also led to a dose dependent increase in ROS production when the cells were co-incubated with *E. coli* (supplementary Fig S8). These actions of sA2MG on human macrophages were abrogated when the cells were pre-incubated with anti-LRP1 antibody (supplementary Fig S8). sA2MG also elicited an increase in bacterial phagocytosis and ROS production with human peripheral blood neutrophils, actions that were sensitive to cell incubation with anti-LRP1 antibody (supplementary Fig S10).

Another protective response to bacterial invasion is the release of bactericidal proteins and peptides that aid in the containment and clearance of the invading pathogen as well as act as signaling molecule guiding the recruitment of leukocytes to the site of inflammation (Wan *et al*, 2011). Incubation of human peripheral blood neutrophils in the presence of LPS led to an increase in cathelecidin levels measured in the cell free supernatants, levels that were further enhanced when the neutrophils were also incubated with sA2MG (supplementary Fig S11). Together these findings suggest that A2MG on microparticles engages LRP1 on human leukocytes to modulate host protective responses.

### Microparticle-A2MG promotes neutrophil – endothelium interactions

**Figure 6:**
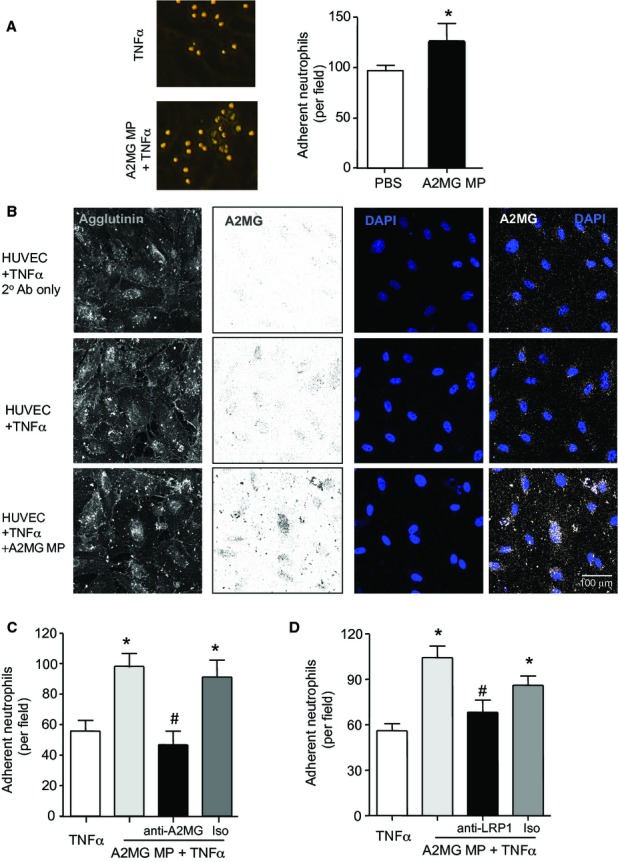
A2MG is transferred by microparticles onto endothelial cell plasma membranes and promotes neutrophil adhesion to endothelial cells. Results are mean ± s.e.m. of *n* = 3–4 distinct microparticle and HUVEC preparations per group (*P < 0.05, versus TNF-α; ^#^*P* < 0.05 versus A2MG MP + TNF-α by Student's *t*-test or one way ANOVA). A  HUVEC were incubated with TNF-α (10 ng/ml) in the presence or absence of microparticles expressing elevated A2MG levels (A2MG MP) for 4 h at 37°C. Freshly prepared neutrophils were then perfused over the monolayer at 1-dyne/cm^2^ for 8 min and the number of adherent leukocytes quantified. B  HUVEC were stimulated with TNF-α (10 ng/ml) with or without A2MG MP (5 × 10^4^) for 4 h, these were then fixed, stained using Alexa Fluor® 633-Agglutinin (Membrane), anti-A2MG antibody (5 μg/ml) and mounted in Probing Antifade medium containing DAPI. C  A2MG MP were incubated with anti-A2MG antibody (5 μg/ml), or isotype control (Iso; 10 min, RT) prior to incubation with HUVEC monolayer (4 h, 37°C), and neutrophil perfusion. D  Neutrophils were incubated with anti-LRP1 (5 μg/ml) antibody or isotype control (Iso; 10 min, RT) prior to perfusion over endothelial cells that were incubated with A2MG MP (4 h, 37°C).

Leukocyte firm adhesion to the vascular endothelium is a key step in regulating recruitment to the site of inflammation (Phillipson & Kubes, 2011), therefore we next investigated whether microparticle-A2MG regulated human neutrophil recruitment onto endothelial cells. Here we incubated (4 h, 37°C) HUVEC with A2MG MP that led to an increase in neutrophil recruitment (≥25% above TNF-α; Fig 6A) onto the endothelial cell monolayer; whereas neutrophil adhesion was unchanged when endothelial cells were incubated with neutrophil microparticles expressing low A2MG levels (*n* = 3).

We next investigated whether A2MG MP regulated endothelial adhesion molecule expression. Flow-cytometric analysis did not reveal any significant differences in expression of levels of CD31, CD54, CD62E, CD99 and CD106 on endothelial cell plasma membranes between cells incubated with PBS and cells incubated with A2MG MP (supplementary Fig S12). Thus, we next investigated whether microparticle-delivered A2MG directly regulated the neutrophil adhesion to endothelial monolayers. Confocal analysis of endothelial cells incubated with A2MG MP demonstrated a marked increase in A2MG immunoreactivity on the HUVEC monolayers (Fig 6B). Therefore we next tested whether this transfer of A2MG by microparticles was of functional relevance incubating A2MG MP with anti-A2MG antibody either prior to incubation with HUVEC or 15 min prior to neutrophil perfusion. In both cases incubation of the microparticles with anti-A2MG antibody abrogated the potentiating actions of A2MG MP on neutrophil adhesion and returned cell adhesion to the values measured following TNF-α stimulation alone (Fig 6C and supplementary Fig S13). Collectively, these results suggest that A2MG is delivered onto the endothelial cells plasma membranes by microparticles where it contributes to neutrophil adhesion to endothelial cells.

We next investigated the role of neutrophil LRP1 in mediating the pro-adhesive actions of A2MG MP. Here incubation of neutrophils with an anti-LRP1 antibody led to a significant reduction in neutrophil adhesion when compared with A2MG MP incubated HUVEC, an effect that was lost when neutrophils were instead incubated with a relevant isotype control (Fig 6D). Together these results indicate that A2MG is transferred by microparticles on to the endothelial plasma membrane where it engages LRP1 on the neutrophils promoting firm leukocyte adhesion.

### A2MG modulates neutrophil responses

**Figure 7:**
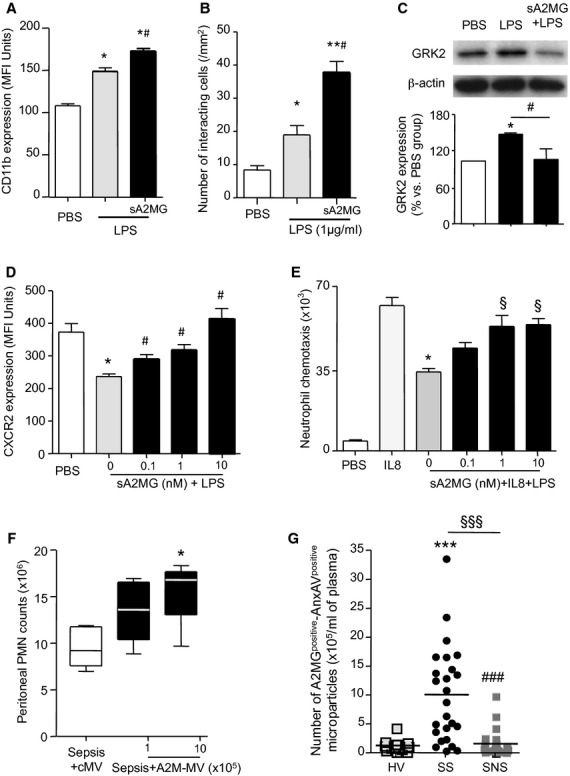
A2MG preserves neutrophil responses in the presence of endotoxin: correlation of A2MG containing microparticles levels with survival in sepsis. A  CD11b neo-epitope exposure following LPS treatment (1 μg/ml, 60 min, 37°C) of isolated human neutrophils was determined by flow-cytometry. B  Neutrophil interaction with ICAM-1 coated chambers following incubation with LPS (1 μg/ml, 60 min, 37°C) or A2MG (10 nM, 15 min, 37°C) followed by LPS (1 μg/ml, 60 min, 37°C) under flow. C  GRK2 expression was assessed by western blotting in human neutrophils incubated with PBS, LPS (1 μg/ml, 60 min, 37°C) or A2MG (10 nM, 5 min, 37°C) followed by LPS (1 μg/ml, 60 min, 37°C). D  CXCR2 expression on human neutrophils incubated with LPS with or without A2MG (60 min, 37°C) as determined by flow cytometry. E  Neutrophil chemotaxis towards IL8 (100 ng/ml) following cell incubation with LPS (1 μg/ml) and A2MG as indicated. Results are mean ± s.e.m. of *n* = 3–4 distinct microparticle and neutrophil preparations (*P < 0.05, **P < 0.001 versus PBS incubations, ^#^*P* < 0.05 versus LPS; ^§^*P* < 0.05 versus IL8+LPS by one way ANOVA). F  Control MV; (10^6^/mouse) or A2MG-MV were administered i.v. at the indicated doses 5 min prior to CLP (see Materials and Methods for details). Mice were sacrificed at 6 h and peritoneal exudate neutrophil counts were determined by light microscopy and flow cytometry. Results are mean ± s.e.m. *n* = 5 mice per group (*P < 0.05 versus MV treated mice by one way ANOVA). G  A2MG surface expression on plasma microparticles (AnxAV^positive^ events) from healthy volunteers (HV; *n* = 15), patients that survive (SS; *n* = 25) and those that do not survive the sepsis (SNS; *n* = 25) as determined by flow-cytometry (see Materials and Methods and supplementary Table S1 for further details; *P < 0.05; ***P < 0.001 versus HV, ^#^*P* < 0.05, ^###^*P* < 0.01 versus SS by one way ANOVA; ^§§§^*P* < 0.01 by two tailed Fisher's exact test).

We further explored the molecular mechanisms by which A2MG regulated neutrophil recruitment to endothelial cells. For this purpose we incubated human neutrophils with sA2MG, eliminating the possibility that other microparticle derived proteins could confound these analyses, and assessed the expression of the active conformation of CD11b, the alpha moiety of Mac-1 that is involved in neutrophil recruitment onto the vascular endothelium (Edens & Parkos, 2003). Neutrophil incubation with sA2MG led to a significant increase in CD11b neo-epitope exposure (Fig 7A). The increased CD11b activation was functionally reflected in an enhanced human and mouse neutrophil interaction with ICAM-1, one of the CD11b ligands (Fig 7B and supplementary Fig S14).

Chemotactic cues direct leukocytes to the site of injury or infection. This response is hampered in sepsis where endotoxin upregulates G-protein-coupled receptor kinase 2 (GRK2) reducing cell surface expression of chemotactic receptors on the neutrophil plasma membrane (Alves-Filho *et al*, 2009) resulting in inefficient neutrophil activation and recruitment (Alves-Filho *et al*, 2009, 2010; Liu *et al*, 2012). Thus, we next investigated whether A2MG can help rectify aberrant neutrophil responses elicited by endotoxin. Incubation of neutrophils with sA2MG inhibited LPS-induced GRK2 upregulation (Fig 7C). This was of functional relevance since sA2M prevented LPS mediated down regulation of CXCR2 (IL-8 receptor) on the neutrophil plasma membrane (Fig 7D) and preserved neutrophil chemotactic responses (Fig 7E).

In order to investigate whether these findings held true *in vivo* we investigated if A2MG regulated early neutrophil recruitment to the site of infection during on-going CLP. Administration of A2MG MV prior to CLP led to a dose-dependent increase in the number of peritoneal exudate neutrophils collected at 6 h when compared to mice treated with MV alone (Fig 7F). Collectively, these results demonstrate the protective actions of A2MG in maintaining key neutrophil responses that are often compromised in sepsis.

### Elevated plasma levels of A2MG expressing microparticles correlate with survival in human sepsis

In view of these potentially clinically important properties displayed by A2MG, especially when incorporated into microparticles, we measured plasma levels of A2MG containing microparticles in patients with sepsis caused by community-acquired pneumonia within 24 h of admission to the intensive care unit (ICU). In order to better appreciate the impact of these microparticles in sepsis we compared survivors (i.e. patients that were alive 28 days post admission to the intensive care unit; *n* = 25) with non-survivors (i.e. patients that died within 28 days of admission to the ICU; *n* = 25). There were no differences in co-morbidities or adjuvant treatments (supplementary Tables S2 and S3) between survivors and non-survivors.

Levels of A2MG containing microparticles were higher in survivors then in both non-survivors and healthy volunteers (HV) as determined by the number of A2MG and Annexin V positive events detected using flow cytometry (Fig 7G). In addition the increase in plasma A2MG containing microparticles was found to be significantly correlated with disease outcome (Fig 7G).

Collectively, these results suggest that microparticle enrichment with A2MG is an endogenous host protective mechanism that preserves key leukocyte responses during sepsis to engage pro-resolving and life saving pathways.

## Discussion

The investigations reported herein demonstrate that A2MG incorporated into microparticles activated distinct host protective responses in murine sepsis, when compared to sA2MG, leading to enhanced bacterial containment and clearance ultimately resulting in improved survival. Microparticle-A2MG was also found to regulate human leukocyte responses, where it promoted neutrophil recruitment to endothelial cells. In addition, A2MG preserved neutrophil chemotactic responses in the presence of endotoxin and stimulated bacterial phagocytosis and ROS production via its receptor, LRP1. These findings support the hypothesis that A2MG incorporation into microparticles may potentiate the protective actions of A2MG in sepsis by activating distinct pro-resolving and tissue protective pathways.

A2MG is an evolutionarily conserved tetrameric glycoprotein (Qin *et al*, 2010), expressed by a number of cell types in its native form and is one of the major circulating anti-proteinases in vertebrates (Rehman *et al*, 2013). Of note A2MG is the only plasma proteinase able to inhibit a wide range of mammalian proteases in addition to parasite-derived proteinases, including those from *Trichophyton mentagrophytes* (causal agent of ringworm) and the neutral proteinase of *Fusiformis nodosus* [causal agent of ovine foot-root; for a detailed review (Rehman *et al*, 2013)]. In mammals, A2MG is predominantly found as a heterotetramer of 160–185 kDa subunits. Upon exposure to proteases or reactive amines A2MG changes conformation, thereby initiating high affinity binding to several cytokines including TNF-α and IL-6. This conformational change also exposes a receptor-binding domain on the protein that leads to its rapid clearance via endocytosis (Imber & Pizzo, 1981). This mechanism has been proposed to mediate the protective actions of A2MG in experimental models of sepsis, by quenching circulating cytokine levels (Webb & Gonias, 1998), in addition to the ability of A2MG to sequester proteinases thereby regulating proteinase activity during inflammatory events (reviewed in Rehman *et al*, 2013).

In other systems A2MG exerts protective actions inhibiting H_2_O_2_ production by polymorphonuclear leukocytes, *Trypanozoma cruzi* induced macrophage and cardiomyocyte apoptosis and clearing unfolded or misfolded proteins from extracellular spaces (reviewed in Rehman *et al*, 2013). Hence, our present findings with A2MG containing microvesicles and in a clinically relevant model of sepsis suggest a novel mechanism of action for A2MG, whereby incorporation of A2MG in microparticles potentiates the endogenous host responses resulting in enhanced protective actions in sepsis when compared to sA2MG.

At the site of inflammation lipid mediators are biosynthesized from polyunsaturated fatty acids and regulate both perpetuation and resolution of inflammation (Spite & Serhan, 2010). Mediators including PG and LT, produced from arachidonic acid, increase vascular permeability and recruitment of leukocytes to the site(s) of inflammation (Flower & Vane, 1974; Samuelsson, 2012). These arachidonic acid derived mediators are also responsible for inflammasome-mediated mortality in experimental models of infection (von Moltke *et al*, 2012). Docosahexaenoic acid derived mediators, such as RvD1 and RvD2, possess potent pro-resolving and tissue protective actions (Spite & Serhan, 2010). These novel lipid mediators also promote bacterial containment and clearance in experimental models of sepsis (Spite *et al*, 2009; Chiang *et al*, 2012). In the present report we found that both sA2MG and A2MG-E microparticle administration led to a significant reduction in exudate pro-inflammatory eicosanoid levels including LTB_4_ and PGD_2_. Of note, only treatment of CLP mice with A2MG-E MP led to a significant increase in exudate levels of the pro-resolving RvD1, RvD5 (Chiang *et al*, 2012) and RvD2 (Spite *et al*, 2009), lipid mediators that stimulate resolution and preserve immune vigilance in murine sepsis. In addition, A2M-E MP administration significantly reduced bacterial loads, both at the site of inflammation and systemically, and protected against hypothermia (Fig 2).

It is noteworthy that sA2MG also displayed mild protective actions *in vivo* although these were to a lesser extent than those displayed by A2M-E MP, especially in terms of survival and regulation of the host responses (Figs 1 and 2). This is in contrast to results obtained when sA2MG was incubated with isolated leukocytes *in vitro* where the free protein displayed biological actions of equal magnitude to those the microparticle bound A2MG (Fig 5 and supplementary Figs S8 and S10). One possible explanation for these differences between *in vivo* and *in vitro* actions for sA2MG is that this protein, in its receptor binding conformation as employed in the current experiments, has a very short systemic half-life, estimated to be of ∼4 min in mice (Imber & Pizzo, 1981) and is primarily cleared by the liver (Shibata *et al*, 2003). Therefore these results underscore the notion that incorporation of this protein into microparticles maximizes the protective actions exerted by A2MG. This notion was further corroborated by the finding that administration of synthetic microvesicles containing A2MG also displayed protective actions in murine sepsis and reduced local and systemic inflammation, as well as bacterial burdens in a dose-dependent manner, ultimately leading to enhanced survival (Figs 3 and 4).

Aberrant leukocyte infiltration in non-target organs and the release of noxious intracellular material is responsible for the development of multiple organ failure in severe sepsis (Qiu *et al*, 2000). A2MG-E MP administration enhanced bacterial containment and clearance, and produced a discreet alteration in systemic (plasma) and local (peritoneal exudate) cytokine profiles, with a reduction in pro-inflammatory cytokine levels, including IL-6, IL-17 and TNF-α. In addition, A2MG-E microparticles augmented local and systemic IFNγ and IL-5 levels. Intriguingly both cytokines have been identified as potential therapeutic interventions for immune compromised patients with sepsis (Docke *et al*, 1997; Linch *et al*, 2012). sA2MG administration only in part replicated the protective actions of A2MG-E MP, with upregulation of exudate IFNγ levels. This specific modulation of local and systemic cytokine levels resulted in a reduction in systemic inflammation and significant protection from secondary organ injury (Fig 2). Collectively, these findings indicate that microparticle-A2MG activated endogenous host protective mechanisms that assure an efficient leukocytic response, mainly involving neutrophils and possibly macrophages, with effective clearance of the pathogen and the timely and efficient onset of resolution, with selective engagement of endogenous resolution pathways, leading to increased survival.

Poor outcomes in sepsis are in many cases attributable to aberrant leukocyte responses that compromise elimination of the infecting pathogen and predispose to recurrent infective episodes (Riedemann *et al*, 2003; Goldenberg *et al*, 2011). It is now appreciated that a compensatory anti-inflammatory response follows, or more likely accompanies, the systemic inflammatory response syndrome (Reddy & Standiford, 2010), perhaps accounting for the failure of classical anti-inflammatory therapies (*vide supra*). Leukocytes provide the first line of defense against invading pathogens, including bacteria. Thus, agents that compromise leukocyte functions hamper the host responses, leading to inadequate containment and uncontrolled systemic inflammation (Alves-Filho *et al*, 2009). A2MG interacts with a member of the lipoprotein receptors, termed LRP1 (Andersen *et al*, 2000) that mediates some of the protective actions displayed by A2MG (Arandjelovic *et al*, 2007). In the present study we found that LRP1 was expressed on both human and mouse neutrophils and mediated the protective actions exerted by A2MG-E microparticles in murine sepsis (Fig 4). In addition LRP1 also mediated the pro-phagocytic and bactericidal actions of A2MG on both neutrophils and macrophages (Fig 5, supplementary Figs S8 and S10). These findings are in accord with the pro-phagocytic actions exerted by A2MG on sheep leukocytes (Murai *et al*, 1996) and underscore the protective role of microparticle-A2MG in preserving leukocyte responses during sepsis (Fig 2).

LRP1 was originally identified as an endocytic receptor; albeit it is now evident that ligand binding to human LRP1 engages specific signaling pathways promoting downstream cellular responses (May *et al*, 2005; Shi *et al*, 2009). In accord with these findings we observed that A2MG promoted exposure of a neo-epitope on the alpha subunit of Mac-1 (CD11b, Fig 7A) and higher neutrophil adhesion to its counter ligand ICAM-1 (Fig 7B and supplementary Fig S13). A2MG was also transferred by microparticles onto the endothelial cell plasma membrane where it promoted neutrophil adhesion. Together these findings suggest a model whereby A2MG deposited onto the endothelial cell plasma membrane can engage LRP1 on the neutrophil leading to the activation of β2-integrins hence neutrophil firm adhesion (Fig 7) via signaling pathways that remain of interest.

At the molecular level, one of the mechanisms responsible for aberrant neutrophil responses involves binding of endotoxin to TLR receptors, resulting in upregulation of GRK2 and the consequent reduction of neutrophil chemotactic receptors, including formyl peptide receptor 1 (Liu *et al*, 2012) and chemokine receptor CXCR2 (Alves-Filho *et al*, 2010). CXCR2 down-regulation leads to insufficient neutrophil recruitment to the site of infection, impaired bacterial containment, uncontrolled systemic inflammation and a poor prognosis (Alves-Filho *et al*, 2010). A2MG prevented endotoxin mediated GRK2 upregulation, CXCR2 down regulation in neutrophils and restored neutrophil chemotactic response to IL-8 (Fig 7). These findings led us to hypothesize that microparticle-A2MG enhanced survival by promoting efficient neutrophil recruitment to the site of infection; a concept that has been postulated for other agents that are protective in sepsis including IL-33 (Alves-Filho *et al*, 2010). This hypothesis was corroborated by the *in vivo* findings with A2MG MV, where administration of these vesicles led to an enhanced and dose dependent increase in early neutrophil recruitment (i.e. at the 6 h interval) to the peritoneum following CLP (Fig 7F).

In conclusion, our findings provide evidence for a novel host protective mechanism in sepsis mediated by A2MG incorporation into microparticles. Microparticle-A2MG is functionally transferred on to endothelial cells plasma membrane where it engages its receptor on the surface of rolling or tethering leukocytes modulating their responses to invading pathogens. In complex on-going systemic response to sepsis, the cellular events instigated by A2MG ultimately lead to efficient disposal of the invading pathogen, upregulation of immunoresolving lipid mediators (e.g. RvD1, and RvD2) as well as immunomodulatory cytokines (e.g. IL-5 and IFN-γ), resulting in significant protection from death in experimental sepsis.

Moreover we recently found that plasma levels of A2MG containing microparticles are elevated in septic patients compared to healthy volunteers (Dalli *et al*, 2013) and here we report that levels of A2MG containing microparticles were significantly elevated in patients that survived sepsis when compared to non-survivors and healthy volunteers (Fig 7F and supplementary Table S1). In view of the increasing incidence of sepsis and the failure of immune suppressive strategies to improve outcome, there is considerable interest in developing novel biomarkers and innovative treatments for this condition (Riedemann *et al*, 2003; Russell, 2006; Goldenberg *et al*, 2011; Wenzel & Edmond, 2012). Also a mounting body of evidence suggests that an alternative approach for the treatment of inflammatory conditions, including sepsis, is to employ endogenous agonists to target host pro-resolution responses (Spite & Serhan, 2010; Levy *et al*, 2012; Ortega-Gomez *et al*, 2013). Our findings provide the rationale for employing A2MG containing microparticles as novel early biomarkers for disease severity and prognosis, for example as noted herein for sepsis. In addition the present findings identify A2MG containing microparticles or microvesicles as a potential therapeutic intervention in patients suffering from severe sepsis/septic shock, especially when targeted to patients with immune compromise and recurrent or persistent sepsis.

## Materials and Methods

Unless otherwise specified, materials were obtained from Sigma-Aldrich Ltd (Poole, UK). Animal work was performed according to Home Office regulations (Guidance on the Operation of Animals, Scientific Procedures Act, 1986) and was approved by the Queen Mary University of London Ethics Committee (London, UK) as well as Harvard Medical School Standing Committee on Animals guidelines for animal care (Protocol 02570). Human cells were prepared according to a protocol approved by the East London & the City Local Research Ethics Committee (no. 06/Q605/40; P/00/029 ELCHA, London, UK). Plasma samples were obtained from patients suffering from severe sepsis/septic shock (defined according to The American College of Chest Physicians/Society of Critical Care Medicine consensus definitions) caused by community-acquired pneumonia (CAP) (defined as a febrile illness associated with cough, sputum production, breathlessness, leukocytosis and radiological features of pneumonia acquired in the community or within <2 days of hospital admission) using a protocol approved by the National Research Ethics Service (NRES Committee South Central-Berkshire, original REC Ref numbers: 05/MRE00/38 and 08/H0505/78). Briefly, 4 ml whole blood was chill centrifuged at 4°C for 10 min at 1600 *g*. Platelet poor plasma was transferred into cryovials and stored at –80°C.

### Microparticle preparation

Human neutrophils were isolated by density centrifugation from whole blood as previously described (Dalli *et al*, 2008). Neutrophils (2 × 10^7^ cells/ml), were either incubated over a HUVEC monolayer for 20 min at 37°C prior to addition of fMLP (1 μM; 20 min at 37°C), yielding micro particles that expressed elevated levels of A2MG (A2MG MP) or incubated with fMLP (1 μM; 20 min at 37°C) in suspension yielding microparticles that expressed low A2MG levels [more details in Dalli *et al* (2013)]. Supernatants were collected and contaminating cells removed by two successive centrifugations (3000 *g*; 10 min at 4°C), prior to pelleting the microparticles by ultracentrifugation (100 000 *g*; 1 h at 4°C) as described (Dalli *et al*, 2008). Microparticle pellets were resuspended with Dulbecco phosphate buffered solution (DPBS) and stored at –80°C prior to further analysis.

Plasma microparticle samples from healthy volunteers or septic patients were prepared by gradient centrifugation and subsequent ultracentrifugation, as describe above. Supplementary Table S1 illustrates the demographic characteristics of the donors. For the septic patients, plasma was obtained within 24 h of admission to the Intensive Care Unit of participating hospitals.

### A2MG microvesicle preparation

A2MG enriched microvesicles (A2MG-MV) were prepared as described in De Cock *et al* (2010) for protein loading in polyelectrolyte multilayer microvesicles. Briefly, vesicles were fabricated by alternating deposition of 7 oppositely charged polymer layers of poly-L-Arginine and dextran sulfate sodium salt with one layer of fluorescently labeled FITC-poly-L-Lysine (all polymers were purchased from Sigma-Aldrich). These alternated layers constituted the microvesicle shell that was shown to be well tolerated *in vivo* studies (De Cock *et al*, 2010). These layers are deposited around co-precipitated CaCO_3_ porous particles with A2MG added at a concentration 0.2 mg/ml according to Petrov *et al* (2005). Multilayer assembly on CaCO_3_ particles with A2MG was followed by the dissolution of calcium carbonate using 0.2 M ethylenediaminetetra-acetic acid. Active A2MG (450 μg; BioMac, Leipzig, Germany), remained entrapped into the microvesicles and A2MG levels were assessed by ELISA (see below for details) as 140 ng/1 × 10^5^ microvesicles. As a control, blank microvesicles (no protein incorporated; cMV) were used, prepared and handled exactly as the A2MG-MV but devoid of any protein, and are termed cMV. The mean diameter of the vesicle was 2–3 μm.

### Flow cytometry and Western Blotting

To determine microparticle levels in healthy volunteer and sepsis patient plasma samples microparticles were double stained using an anti-CD66b PE-conjugated antibody (1:25; clone G10F5; BioLegend, Cambridge UK) and Alexa488 conjugated anti-A2MG (5 μg/ml; Clone 3D1; Thermo Scientific, Northumberland, UK). In all cases, microparticles were incubated with the antibodies or relevant isotype controls for 45 min at 4°C prior to analysis with a FACSCalibur flow cytometer (Becton Dickinson, San Jose, CA, USA) using CellQuestTM software (Becton Dickinson).

Surface expression of adhesion molecules on endothelial adhesion were assessed on HUVEC (passage 1–3) stimulated with TNFα (10 ng/ml; R&D Systems, Abingdon, UK) and incubated with or without A2MG MP (8 × 10^5^/9.6 cm^2^/well) for 4 h at 37°C. Expression of CD62E/P (clone: 1.2B6), CD106 (clone: 1.G11B1), CD31 (clone: WM59) CD54 (clone: HA58), CD99 (clone: DN16) or A2MG (clone: 257316) were determined by flow cytometry following standard protocols.

A2MG, in its active conformation (cat no: 05-04; BioMac), was always employed when added exogenously. Human neutrophils were incubated with LPS (1 μg/ml) following a 5-min pretreatment with vehicle (PBS) or active A2MG. For assessment of CD11b neo-epitope exposure, anti-CD11b FITC-conjugated antibody (clone: CBRM1/5) was added to the neutrophils in suspension prior to LPS stimulation (10 min, 37°C). Subsequently, cells were washed and staining assessed as above. In a separate set of experiments neutrophils were pre-incubated with A2MG (0.1–10 nM, 5 min), then stimulated with LPS (strain 055B:B5 Sigma; 1 μg/ml, 60 min), prior to washing and assessment of CXCR2 levels using a mouse anti-human CXCR2 PE-conjugated antibody (clone: 5E8; Biolegend).

In some experiments human neutrophils were incubated with or without A2MG (10 nM, 5 min, 37°C) prior to LPS stimulation (1 μg/ml, 60 min, 37°C) and GRK2 expression assessed by Western blotting (clone:sc-562; 0.5 μg/ml). Proteins were detected using an ECL detection reagent and visualized on HyperfilmTM (GE Healthcare, Buckinghamshire, UK).

Human peripheral blood neutrophils were incubated with PBS or LPS (1 μg/ml, 1 h, 37°C). Cells were then either taken for staining or permeabilized using a Cytofix/Cytoperm buffer solution (BD Biosciences, San Jose, CA, USA) prior to staining. Next the neutrophils incubated with anti-LRP1 antibody (5 μg/ml; clone A2MGr alpha-2; Invitrogen, Paisley, UK) for 45 min or relevant isotype control and then with an anti-mouse Alexa Fluor® 488 conjugated antibody (10 μg/ml; Invitrogen) for 45 min. LRP1 levels are expressed in MFI units relative to Isotype control.

Bacterial phagocytosis in murine inflammatory exudates was assessed by flow cytometry as in Vaishnava *et al* (2011) with some modifications. Peritoneal exudates were placed in 96 well plates washed to removed any unphagocytosed bacteria. The cells were then fixed and permeabilized using Cytofix/Cytoperm buffer solution (BD Biosciences) for 30 min at 4°C. The cells were the incubated with a solution containing 0.1 mg/ml lysozyme, 100 mM Tris–HCl and 50 mM EDTA for 5 min on ice. The cells were then washed with buffer and incubated with 25 μM of bacterial specific probe (sequence: GCTGCCTCCCGTAGGAGT) or a non-specific control probe (sequence: ACTCCTACGGGAGGCAGC) conjugated to Alexa Fluor® 488 (Operon, Alameda, CA, USA) and incubated overnight at 50°C. The cells were then washed 3× in wash buffer and the extent of phagocytosis was determined by flow cytometry.

### Flow chamber

To assess leukocyte-endothelial interactions, HUVEC were plated in μ-Slides VI0.4 (Ibidi, Munchen, Germany). Confluent monolayers were stimulated with TNF-α (10 ng/ml, 4 h; R&D systems) and incubated with or without A2MG MP (5 × 10^4^/0.6 cm^2^ channel) for 4 h at 37°C. In determinate experiments, A2MG MP were pre-incubated for 15 min with anti-A2MG (5 μg/ml) prior to addition to HUVEC for 4 h, or antibodies were added to HUVEC only 15 min prior to flow (that is the last 15 min of the 4 h incubation with A2MG MP). Human neutrophils (1 × 10^6^/ml) were perfused over HUVEC at 1 dyne/cm^2^ for 8 min, then six random fields were recorded for 10 s and images analysed using Image ProPlus 7 (MediaCybernetics, Bethesda, MD, USA). Neutrophils that remained stationary were quantified as adherent.

In a separate set of experiments μ-Slides VI0.4 (Ibidi) were coated using hrICAM-1 (50 μg/ml; R&D Systems) for 60 min at room temperature. Human peripheral blood neutrophils isolated as described above were pre-incubated for 5 min with vehicle (PBS) or A2MG (10 nM), subsequently stimulated with LPS (1 μg/ml, 60 min) and perfused over the ICAM-1 coated chamber at 1 dyne/cm^2^ for 8 min.

In a separate set of experiments μ-Slides VI0.4 (Ibidi) were coated using rmICAM-1 fc chimera (12.5 μg/ml; R&D Systems) for 60 min, then blocked with 1% BSA for 30min. Murine blood was collected in sodium citrate (1:10, 3.2%) and diluted 1:10 in HBSS. Blood was treated for 15 min with vehicle (PBS) or A2MG (10 nM), subsequently stimulated with LPS (1 μg/ml, 60 min, 37°C) and flowed over the ICAM-1 coated chamber at 1 dyne/cm^2^ for 8 min.

### Confocal microscopy

Neutrophils were assessed for surface expression of A2MG receptor (LRP1) along with the lineage specific lineage marker (CD11b) under resting conditions and after LPS incubation (1 μg/ml, 1 h, 37°C). Cells were labeled with anti-LRP1 unconjugated antibody (5 μg/ml; clone A2MGr alpha-2; Invitrogen) for 45 min and then with specific secondary antibody Alexa Fluor® 488 (10 μg/ml; Invitrogen) for 45 min. Finally, anti-CD11b APC-conjugated antibody (2 μg/ml; clone ICRF44; eBioscence, Hatfield, UK) was added together with the relevant isotype controls, for 1 h at 4°C. Cells were then washed with PBS and re-suspended in deionized H_2_O and transferred onto on microscope slides and mounted in Probing Antifade medium (Invitrogen) containing DAPI. The staining was visualized using a Zeiss LSM 510 META scanning confocal microscope (×63 oil-immersion objective) and analysed by Zeiss LSM Imaging software (Carl Zeiss, Oberkochen, Germany).

HUVEC were plated in μ-Slides VI0.4 (Ibidi), and confluent monolayers were stimulated with TNF-α (10 ng/ml) in presence or absence of A2MG^high^ MPs (5 × 10^4^) for 4 h (37°C). HUVEC were then fixed with 1% paraformaldehyde (PFA; 10 min, 4°C). Cells were stained with Alexa Fluor® 633-Agglutinin (1 μg/ml; Invitrogen; 20 min, RT) and then blocked for 30 min in PBS containing 5% FBS. Unconjugated primary anti-A2MG antibody (5 μg/ml; R&D System) was then added for 1 h followed by Alexa Fluor® 594 secondary antibody (5 μg/ml; Invitrogen) for 45 min. Cells were washed in PBS, mounted and imaged as above.

### Microparticle A2MG-enrichment

Microparticles were obtained from human neutrophils as described above. Intercalation of active A2MG (0.1 mg per 5 × 10^5^ microparticles) with fluorescent phospholipid (1,2-dioleoyl-sn-glycero-3-phoshpo-L-serine-N-7-nitro-2-1,3-benzoxadiazol-4-yl; 100 mg; Avanti Polar Lipids, Alabaster, AL, USA) was performed by aqueous energy dissemination using a sonic dismembrator (output power 15 W, 15 min, 25°C; Fisher Scientific, Loughborough, UK) as previously described (Norling *et al*, 2011) (supplementary Fig S1). A2MG-E microparticles were then ultra-centrifuged at 100 000 *g* to remove any unbound protein and resuspended in 0.2-μm filtered PBS. Incorporation of A2MG was confirmed by flow cytometry (BD FACSCanto II; BD Biosciences) staining with an isoform specific anti-A2MG antibody (Clone a1/IIE7; BioMac).

### Coecal ligation and puncture

Coecal ligation and puncture (CLP) was performed in male FVB mice (5–6 weeks old). The cecum was ligated below the ileo-caecal valve to produce severe sepsis (Rittirsch *et al*, 2009). A through and through puncture was performed with a 20 gauge needle, followed by one additional puncture in the distal tip of the cecum. Mice received saline (500 μl s.c.) followed by i.v. administration of 100 μl of PBS, sA2MG (0.05 μg) or A2MG-E microparticles (1 × 10^5^) 1 h after closure of the peritoneum. Animal survival was followed up to day 4. In separate experiments, mice were treated as above, with control microvesicles (cMV; 10^6^/mouse) or A2MG-MV at the indicated concentrations and sacrificed at 6 h or 12 h post CLP. Body temperature was measured with a thermal probe (FLUKE Corporation, Everett, WA, USA). Blood was collected by cardiac puncture, peritoneal lavage fluid and lung tissue were also collected. Blood and peritoneal bacterial loads were determined by growth on tryptic soy agar plates. Plasma and peritoneal cytokine levels were determined by Multiplex array. Peritoneal cells were counted using Turks solution and differentiated using anti-F4/80 (Clone BM8; BioLegend) and anti-Ly6G (Clone 1A8; BioLegend) antibodies. Neutrophil infiltration within the lungs was assessed following mechanical disruption of the tissue and staining for anti-Ly6G.

Using *in vivo*-JetPEI™ (Polyplus, Illkirch, France) transfection reagent as a carrier for delivering plasmids *in vivo* we administered 30 μg of mock or mouse LRP1 siRNA lentiviral vectors (Applied Biological Materials Inc., Richmond, VA, USA). The plasmid containing solution was prepared following manufacturer's instructions and the plasmid was delivered by i.v. injection. LRP1 expression in peripheral blood was determined after 48 h by realtime PCR (see details below).

### Targeted LC-MS/MS-based lipidomics of CLP peritoneal exudates

Exudate lavages were collected and immediately added to two volumes of cold methanol for extraction and work-up as in (Yang *et al*, 2011). LC-MS-MS identification was acquired with an Agilent 1100 series HPLC (Santa Clara, CO, USA) paired with an ABI Sciex Instruments (Framingham, MA, USA) 5500 Q-TRAP linear ion trap quadrupole mass spectrometer. The column (Agilent Eclipse Plus C18, 4.6 mm × 50 mm × 1.8 μm) was eluted at a flow rate of 0.4 ml/min with methanol/water/acetic acid (60/40/0.01; v/v/v) ramped to 80/20/0.01 (v/v/v) after 5 min, 95/5/0.01 (v/v/v) after 8 min, and 100/0/0.01 after 14 min to wash the column. Instrument control and data acquisition were performed using Analyst 1.5 software (ABSciex, Framingham, MA, USA). Ion pair transitions from previously reported multiple reaction monitoring (MRM) methods were used for profiling and quantitation of RvD1 (375.2/233.1) RvD2 (375.2/175.1), PD1 (359.2/153.1), PGD_2_ (351.2/189.1), PGE_2_ (351.2/233.1), PGF_2α_ (353.2/193.1) and LTB_4_ (335.2/195.1). Criteria for identification were: LC retention time and a minimum of six fragment diagnostic ions on the MS/MS spectrum matching those of synthetic standards. Deuterated 5*S*-HETE, PGE_2_ and LTB_4_ (Cayman Chemicals, Ann Arbor, MI, USA, 500 pg) were added prior to extraction as internal standard for recovery calculations.

### Gene expression

Peripheral blood was collected in heparinized syringes from tail veins of mice that received either mock vector or siRNA carrying vectors after 48 h. Blood was immediately placed in TRIzol® (Invitrogen, New York, NY, USA) and RNA was extracted following manufacturer's instructions. RNA quantity and quality was assessed on a NanoDrop 2000 (ThermoScientific, Wilmington, DE, USA). cDNA was obtained using a SuperScript® III First-Strand Synthesis SuperMix and sequence specific primers for mouse GAPDH and LRP 1 following manufacturer's instructions. Realtime PCR was performed using RT^2^ mastermix (Qiagen, Manchester, UK) following manufacturer's instructions and loading ∼100 ng of cDNA per well.

### *E. coli* phagocytosis and generation of intracellular reactive oxygen species

Human neutrophils and monocyte derived macrophages were adhered to plastic and pre-incubated with A2MG in the activated conformation (1–100 nM), A2MG MP (10^2^–10^5^) or vehicle for 15 min prior to incubation with pre-labeled *E. coli* (O6:K2:H1; 50:1 ratio, BacLight) 37°C, 60 min. Non-phagocytosed bacteria were washed off and extracellular fluorescence quenched using Trypan blue (1:50 dilution). The number of phagocytosed cells was assessed using a Spectra Max M3 (Molecular Devices, Sunnyvale, CA, USA) plate reader by measuring fluorescence emission at 525 nM.

In separate experiments human neutrophils and monocyte derived macrophages were incubated with CM-H_2_DCFDA (5 μM) for 30 min prior to washing and plating on a 96 well plate. Nonadherent cells were removed and cells incubated A2MG in the activated conformation (1–100 nM), A2MG MP (10^2^–10^5^) or vehicle for 15 min prior to incubation with *E*. *coli* (O6:K2:H1; 50:1 ratio) for an additional 30 min at 37°C. Probe oxidation was determined using a fluorescent plate reader.

### Neutrophil chemotaxis

Human neutrophils were isolated as detailed above and resuspended at 4 × 10^6^/ml in PBS containing 0.05% BSA. These were then incubated with either PBS or A2MG for 15 min prior to incubation with LPS (1 μg/ml) for 15 min. Chemotaxis towards IL8 (100 ng/ml) was then assessed using Neuroprobe 96 well ChemoTx® chambers with 3 μM pore size over 90 min.

### A2MG ELISA

Standards (0.005–5 μg/ml), A2M-E MP or A2MG microparticles were transferred in 100 μl to a 96 well high binding plate (Corning® Costar®; Sigma-Aldrich) and incubated overnight at 4°C. Plates were washed three times and blocked using PBS containing 2% BSA (RT). After 1 h plates were washed and incubated with anti-A2MG (1:50 000; BioMac) for 2 h (RT). Plates were then washed and incubated with anti-mouse HRP conjugated antibody (1:5000; Invitrogen) for 2 h at RT. Subsequently plates were washed and incubated with TMB substrate buffer (R&D System) for 30 min at RT. The reaction was stopped with 1 N sulphuric acid and absorbance at 450 nm was measured on a plate reader.

### Statistical analyses

Experiments were performed in triplicate and results expressed as Mean ± s.e.m. Statistical differences were determined using one-way analysis of variance or two-tailed unpaired Student's *t-*test as appropriate. Kaplan–Meier survival curves were analyzed using a one-tailed log-rank test using GraphPad Prism™ (GraphPad Software, La Jolla, CA, USA). A *P* value < 0.05 was taken as significant for rejection of the null hypothesis.

**The Paper Explained**

**Problem**

Sepsis is a medical condition resulting from invasive infection that leads to whole-body inflammation. This is partly a result of the inability of the body's immune cells to effectively eliminate the pathogenic organisms leading to wide spread infection that can result in death. Current treatments, including the administration of antibiotics and anti-inflammatory drugs, have been largely ineffective; therefore there is a significant need to develop more effective treatments for this condition.

Recently we found that a specific subset of microparticles enriched in alpha-2-macroglobulin (A2MG) are increased in the plasma of sepsis patients when compared to healthy volunteers. Microparticles are small membrane bound vesicles implicated in the regulation of inflammation, and its timely resolution. They do this by transporting proteins and other mediators from the cell of origin to its destination, thereby acting as an endogenous delivery system for these signaling molecules.

**Results**

In the present study we investigated the actions of microparticles enriched in A2MG in regulating the body's immune responses during sepsis. We found that these microparticles protected septic mice from dying and improved the containment and clearance of bacteria in these mice. A2MG enriched microparticles also stimulated the production of mediators at the site of inflammation that are known to promote the clearance of bacteria and resolution of sepsis. The actions of these microparticles were at least in part mediated by enhancing the ability of white blood cells to respond to bacterial signals. We also found that plasma levels of A2MG containing microparticles were higher in septic patients who survived than in those who died.

**Impact**

Our results provide the rationale for employing A2MG containing microparticles as novel early biomarkers for disease severity and prognosis in sepsis. In addition, we provide evidence for the potential of A2MG containing microparticles as a novel treatment for patients suffering from severe sepsis/septic shock.

## Supplementary information

for this article is available online: http://embomolmed.embopress.org
