# Supplementary material for: Microparticle alpha-2-macroglobulin enhances pro-resolving responses and promotes survival in sepsis
Source: EMBO Mol Med. 2013 Dec 16;6(1):27–42. doi: 10.1002/emmm.201303503 (PMC3936490; doi:10.1002/emmm.201303503)
Supplement: Supplementary file 1 [file emmm0006-0027-sd1.pdf]

EMBO Molecular Medicine

Microparticle Alpha-2-Macroglobulin Enhances Pro-resolving Responses and Promotes Survival in Sepsis

Jesmond Dalli, Lucy V Norling, Trinidad Montero-Melendez, Donata Federici Canova, Hazem Lashin, Anton M Pavlov, Gleb B Sukhorukov, Charles J Hinds and Mauro Perretti

Corresponding author: Mauro Perretti, Barts and The London School of Medicine and Dentistry

Review timeline:

Submission date:	29 May 2013
Editorial Decision:	14 June 2013
Additional Author Correspondence :	16 June 2013
Additional Editorial Correspondence :	17 June 2013
Resubmission:	18 September 2013
Editorial Decision:	26 September 2013
Revision received:	02 October 2013
Accepted:	04 October 2013

Transaction Report:

(Note: With the exception of the correction of typographical or spelling errors that could be a source of ambiguity, letters and reports are not edited. The original formatting of letters and referee reports may not be reflected in this compilation.)

Editor: Céline Carret

1st Editorial Decision 14 June 2013

Thank you for the submission of your manuscript to our editorial office. We have now received feedback from 2 reviewers whom we asked to evaluate your manuscript. Given that both reviewers provide very similar recommendations, we prefer to make a decision now in order to avoid further delay in the process.

As you will see from the enclosed reports, neither referee supports publication of the manuscript in EMBO Molecular Medicine. Although they do acknowledge the potential interest of the topic, they also feel that the data provided is not sufficient to draw firm conclusions. I would prefer not to repeat their individual points of criticism in this letter, but clearly both of them point to a number of serious conceptual and technical shortcomings of your study, and feel that given these limitations, your results would appear rather preliminary.

Given these negative opinions and the fact that EMBO Molecular Medicine can only afford to accept papers which receive enthusiastic support from a majority of referees, I am afraid that I see little choice but to return the manuscript to you at this point with the decision that we cannot offer to publish it.

I am sorry to have to disappoint you in this matter.

***** Reviewer's comments *****

Referee #1 (Remarks):

Dalli and colleagues investigate the impact of A2MG-enriched microparticles in a mouse model of polymicrobial sepsis- They evidence a protective effect which may mechanistically be explained by changes (i) in lipid-mediator and cytokine profile (ii) in neutrophil and macrophages to phagocytose bacteria and (iii) in neutrophil adhesion. Although the entire concept is very interesting and novel, I have major questions regarding study design, controls and some of the methods.
1. Figure 1: More control groups are required in B. What is the effect of a scrambled version of A2MG or of A2MG-null microparticles?
2. Figure 2: It appears that soluble A2MG promotes survival (s. Fig. 1) but does not share the same mechanisms with regard to bacterial clearance and leukocyte accumulation as compared to A2MG-enriched microparticles. How can this be explained? How are circulating WBC affected by the treatment with A2MG-E or sA2MG? What is the effect of the treatments on monocyte/macrophage accumulation?
3. Figure 3: Is LRP1 pre-formed and mobilized or de-novo produced in response to LPS? Does human A2MG also interact with mouse LRP1 and result in enhanced bacterial uptake? The increase in uptake is rather modest. Hence, the question arises to what extend this mechanism contributes to the in vivo effects in Fig. 1 and 2. Does A2MG affect other antimicrobial effector mechanisms, such as ROS production, NET release or release of antimicrobial polypeptides?
4. Figure 4: How does the effect observed here relate to the in vivo situation? There, A2MG delivery reduces neutrophil accumulation, whereas the in vitro data seem to suggest the opposite. What is the effect of an antibody to LRP1 in the adhesion assays?

Referee #2 (Remarks):

In this paper, the authors describe that the acute phase reactant alph-2-macroglobulin (A2MG) has therapeutic effects in sepsis, especially the A2MG that is found bound to microparticles. The data that are presented are of value, but they are too premature to lead to solid conclusions. The main flaws of the paper are the following:

1. In the in vivo experiments (CLP model in mice), a number of essential controls are lacking. It is important that empty microparticles are involved in the study, as well as microparticles uploaded with other proteins than A2MG. Also a comparison with sA2MG can only be made if the correct amount of protein is known that is delivered with the A2MG-E MP.

2. In most of the experiments, just one dose of A2MG is used. It is important, in vivo and in vitro, to study dose-repsonse effects in order to be able to draw conclusions.

3. The basic observations in the mouse CLP model raise interest with the reader, but most of the mechanistic investigations are then done in human cells. All of the in vitro studies should also be peformed in mice, for obvious reasons.

4. Many of the data displayed in the figures are hard to understand because they are not properly explained in the paper (results, materials or legends). For example, Fig 1A and 1C.

5. Some of the effects of A2MG are blocked by inhibiting the receptor of A2MG, namely the LRP. It is important that as many as possible effects of A2MG are investigated in conditions of LRP inhibition, knockout or knockdown.

6. Since there appear to be differences in effects between A2MG-E microparticles and free sA2MG, it is confusing why in certain experiments only sA2MG is used. There should be better motivation for this choice.

More background on A2MG should be proved in the paper.

Additional Author Correspondence 16 June 2013

Thank you for your June 14th email that, despite the negative outcome, contained encouraging comments. We thank the Reviewers and your office for appreciating the novelty and the potential implications of our current work.

We have studied the comments raised by the Reviewers and, in essence, we agree with (most of) them. Since EMM is our preferred target for this work, in view of its status and recent high profile study on microparticles (Cloutier et al EMBO M.M. 2012), we would like to inquire whether you will consider a revised version of the manuscript where we address the points raised by the reviewers. In particular we will perform additional CLP experiments with different doses of A2M incorporated into microparticles as well as with an empty microparticles and assess the role of the LRP1 receptor in additional experimental settings as requested by the Reviewers.

We look forward to hearing your view.

Additional Editorial Correspondence 17 June 2013

Thank you for your letter. I appreciate your interest in EMBO Molecular Medicine.

Should you be able to address all comments raised by referees with additional experiments when needed and support all conclusions by experimental results, we would be willing to reconsider the manuscript as an invited re-submission. However, I should say that the manuscript will be editorially evaluated afresh in terms of recent literature etc, and it does not mean that we will send it back to referees. Nevertheless, if we do, and to facilitate the job of the reviewers, please provide a point-by-point letter with your resubmission.

Best of luck with the forecoming experiments.

Resubmission 18 September 2013

Referee #1 (Remarks):

Dalli and colleagues investigate the impact of A2MG-enriched micro particles in a mouse model of polymicrobial sepsis- They evidence a protective effect which may mechanistically be explained by changes (i) in lipid-mediator and cytokine profile (ii) in neutrophil and macrophages to phagocytose bacteria and (iii) in neutrophil adhesion. Although the entire concept is very interesting and novel, I have major questions regarding study design, controls and some of the methods.

Thank you for appreciating the novelty of the study, from its conception to the data presented and their implications.

1. Figure 1: More control groups are required in B. What is the effect of a scrambled version of A2MG or of A2MG-null micro particles?

We agree and have now addressed this in the revised manuscript by constructing synthetic vesicles that are loaded with A2MG and investigated their actions with respect to vesicles that were not loaded with proteins, thereby allowing us to directly investigate the actions of micro vesicle-

incorporated A2MG. These results are now reported in Figure 4A and described on page 8 last three lines and page 9 first line as follows:

"Administration of A2MG-MV ($1x10^6$) led to a significant reduction in mortality when compared with mice treated with cMV (Figure 4A). Together these results indicate that A2MG exerts protective actions in sepsis likely mediating at least some of the protective actions displayed by A2MG-E MP (Figures 1 and 2)."

2a). Figure 2: It appears that soluble A2MG promotes survival (s. Fig. 1) but does not share the same mechanisms with regard to bacterial clearance and leukocyte accumulation as compared to A2MG-enriched micro particles. How can this be explained?

We thank the reviewer for raising this important point. We have now added a detailed explanatory paragraph in the revised manuscript, Discussion page 17 second paragraph that reads as follows:

"It is noteworthy that sA2MG also displayed mild protective actions in vivo although these were to a lesser extent than those displayed by A2M-E MP, especially in terms of survival and regulation of the host responses (Figure 1 and 2). This is in contrast to results obtained when sA2MG was incubated with isolated leukocytes in vitro where the free protein displayed biological actions of equal magnitude to those the micro particle bound A2MG. One possible explanation for these apparent differences between in vivo and in vitro actions for sA2MG is that this protein, in its receptor binding conformation as employed in the current experiments, has a very short systemic half-life in the circulation, estimated to be of ~4 min in mice (Imber & Pizzo, 1981) and is primarily cleared by the liver (Shibata et al, 2003). Therefore these results underscore the notion that incorporation of this protein into micro particles maximizes the protective actions exerted by A2MG. This notion was further underscored by the finding that administration of synthetic micro vesicles containing A2MG also displayed protective actions in murine sepsis and reduced local and systemic inflammation, as well as bacterial burdens in a dose-dependent manner, ultimately leading to enhanced survival (Figure 3 and 4)."

2b) How are circulating WBC affected by the treatment with A2MG-E or sA2MG?

We have now performed additional experiments to address this point observing elevated WBC counts upon treatment. The results from these analysis are presented in Supporting information Figure 2A and discussed on page 7 line 12-14 and page 7 last line that read:

(page 7, lines 14-18)

"Assessment of circulating white blood cell count demonstrated an increase in circulating leukocyte levels following A2MG-E micro particle administration (Supporting Information Figure 4C). This is in keeping with a reduction in secondary organ injury and thus a reduction in neutrophil trafficking to non-infected organs, a hallmark of sepsis (Qiu et al, 2000)."

(page 7, last line)

"In addition there were no differences in the number of circulating leukocytes when compared to PBS treated mice (Supporting Information Figure 4C)."

2c) What is the effect of the treatments on monocyte/macrophage accumulation?

We have now performed additional experiments to address this point. At the time points investigated, reductions in neutrophils were not matched by changes in monocyte and macrophage counts, yielding a favourable ratio. The results from these analysis are presented in Supporting information Figure 2A and discussed on page 7 page lines 2-7 and page 7 last 3 lines read:

(page 7, lines 2-7)

"Of note this reduction in leukocytes was primarily due to a significant reduction in neutrophils but not monocytes/macrophages (Supporting information Figure 4A) leading to a significant increase in the monocyte/macrophage to neutrophil ratio (Supporting information Figure 4B), a hallmark of resolution (Chiang et al, 2012; Spite et al, 2009). Assessment of exudate cytokine levels demonstrated a significant decrease in pro-inflammatory cytokines including IL-1β (~80%, $p<0.05$) and TNF-α (~50%, $p<0.05$; Supporting Information Figure 2C) in A2MG-E micro particle treated animals."

(page 7, last 3 lines)

"We also observed a significant reduction in monocyte/macrophage levels in the CLP exudates when compared to both PBS and A2MG-E micro particles treated mice (Supporting Information Figure 4A)."

<u>*3a). Figure 3: Is LRP1 pre-formed and mobilized or de-novo produced in response to LPS?*</u>

We have now conducted additional experiments to address this point. We detected LRP1 immunoreactivity in mouse and human neutrophils, with a higher expression in the cytosolic compartment of resting cells. In line with many other proteins, this pool may be readably mobilised upon cell activation, as was observed neutrophil incubation with LPS. These results are displayed in Supporting Information Figure 7 and discussed on page 9 line 20-26 lines and page 10 lines 1-6 that read:

"We used flow cytometry to assess the relative distribution of LRP1 on human neutrophils. Here we found that LRP1 immunoreactivity was higher in permeabilized cells in comparison to non-permeabilized cells (Supporting information Figure 7A) demonstrating that this protein was expressed both on the plasma membrane and, at higher levels, in the cytosol. Confocal analysis corroborated LRP1 expression on the plasma membrane of resting human neutrophils (Figure 5A). Assessment of LPR1 expression following exposure to LPS demonstrated a significant upregulation of LRP1 expression on human neutrophils plasma membrane (Figure 5A and Supporting Information Figure 7B). Incubation of mouse neutrophils with LPS also led to a significant increase in cell membrane LRP1 expression (Supporting Information Figure 7C). These results suggest that neutrophil activation leads to LRP1 mobilization from intracellular stores to the plasma membrane."

<u>*3b) Does human A2MG also interact with mouse LRP1 and result in enhanced bacterial uptake?*</u>

We have now conducted additional experiments to investigate whether human A2MG regulated mouse leukocyte responses. The results of this analysis are reported in Supporting Information Figure 9 and Figure 13 and discussed on page lines 10 lines 18 to 22 and page 12 last paragraph and page 13 lines 1-5 that read:

(page 10, lines 18-22)

"In order to assess the role of A2MG in regulating the leukocyte responses elicited by A2MG MP we next investigated whether sA2MG regulated macrophage and peripheral blood neutrophil phagocytosis and ROS production. Incubation of human and mouse macrophages with sA2MG led to a dose dependent increase in macrophage phagocytosis (Supporting Information Figure 8 and 9)."

(page 12, last paragraph to page 13 lines 1-5)

"We further explored the molecular mechanisms by which A2MG regulated neutrophil recruitment to endothelial cells. For this purpose we incubated human neutrophils with sA2MG, eliminating the possibility that other micro particle derived proteins could confound analyses, and assessed the expression of the active conformation of CD11b, the alpha moiety of Mac-1 that is involved in neutrophil recruitment onto the vascular endothelium (Edens & Parkos, 2003). Neutrophil incubation with sA2MG led to a significant increase in CD11b neo-epitope exposure (Figure 7A). The increased CD11b activation was functionally reflected in an enhanced human and mouse neutrophil interaction with ICAM-1, one of the CD11b ligands (Figure 7B, Supporting Information Figure 14)."

3c) The increase in uptake is rather modest. Hence, the question arises to what extend this mechanism contributes to the in vivo effects in Fig. 1 and 2. Does A2MG affect other antimicrobial effector mechanisms, such as ROS production, NET release or release of antimicrobial polypeptides?

We thank the reviewer for raising this point. We have now conducted additional in vivo experiments with mice and in vitro experiments with human leukocytes, monitoring phagocytosis and ROS generation by neutrophils and macrophages as well as cathelicidin release by neutrophils. In addition we also bacterial phagocytosis in vivo using an ex vivo *in situ* hybridization protocol and flow cytometry. These results are now presented in Figure 3B, Figure 5C, E and Supporting Information Figures 8, 10, and 11 described in the text on page 8 lines 13-18, page 10 line 7 to page 11 line 12 that read:

(page 8, lines 13-18)

"Treatment of mice with A2MG-MV also led to a dose-dependent increase in the number of phagocytosed bacteria in murine peritoneal exudates, as determined by in situ hybridization and flow cytometery (Figure 3B). Furthermore, there was a dose dependent reduction in both exudate (Figure 3C) and plasma (Figure 3D) bacterial loads when compared to mice treated with cMV.."

(page 10 line 7 to page 11 line 12)

"Next we investigated the role of LRP1 in mediating the protective responses of microparticle-A2MG on human primary leukocytes. Incubation of neutrophils with micro particles containing elevated A2MG levels, (termed A2MG MP; see methods and (Dalli et al, 2013) for details) led to a dose dependent increase in bacterial phagocytosis (Figure 5B), an action that was inhibited when leukocytes were pre-incubated with an anti-LRP1 antibody. When human neutrophils were incubated with A2MG MP we found a significant increase in ROS production when the neutrophils were also incubated with E. coli (Figure 5C), an action inhibited when cells were pre-incubated with an anti-LRP1 antibody. Similar results were obtained with macrophages both in terms of bacterial phagocytosis (Figure 5D) and ROS production (Figure 5E). These results indicate that leukocyte LRP1 mediates the protective actions of A2M MP.

In order to assess the role of A2MG in regulating the leukocyte responses elicited by A2MG MP we next investigated whether sA2MG regulated macrophage and peripheral blood neutrophil phagocytosis and ROS production. Incubation of human and mouse macrophages with sA2MG led to a dose dependent increase in macrophage phagocytosis (Supporting Information Figure 8 and 9). Incubation of human macrophages with sA2MG also led to a dose dependent increase in ROS production when the cells were co-incubated with E. coli. (Supporting Information Figure 8). These actions of sA2MG on human macrophages were abrogated when the cells were pre-incubated with anti-LRP1 antibody (Supporting Information Figure 8). sA2MG also elicited an increase in bacterial phagocytosis and ROS production with human peripheral blood neutrophils actions that were sensitive to cell incubation with anti-LRP1 antibody (Supporting Information Figure 10).

Another protective response to bacterial invasion is the release of bactericidal proteins and peptides that aid in the containment and clearance of the invading pathogen as well as act as signalling molecule guiding the recruitment of leukocytes to the site of inflammation (Wan et al, 2011). Incubation of human peripheral blood neutrophils in the presence of LPS led to an increase in cathelecidin levels measured in the cell free supernatants, levels that were further enhanced when the neutrophils were also incubated with sA2MG (Supporting Information Figure 11). Together these findings suggest that A2MG on micro particles engages LRP1 on human leukocytes to modulate host protective responses."

4a). Figure 4: How does the effect observed here relate to the in vivo situation?

Please see response to point 3 above.

4b). There, A2MG delivery reduces neutrophil accumulation, whereas the in vitro data seem to suggest the opposite.

We thank the reviewer for raising this important point. We have conducted additional experiments to address this these different actions. In vivo A2MG-E MP or A2MG-MV accelerate the immune response by efficient leukocyte recruitment and disposal of bacteria: this would mean a more rapid instauration of resolution, hence some of the inflammation/disease markers are improved upon administration of these treatments. Monitoring the markers of inflammation at 6 h post-CLP demonstrated that the increased neutrophil recruitment occurs in vitro also occurs during the early stages of the inflammatory response in vivo that may lead to the observed efficient bacterial containment and clearance. These results are displayed in Figure 7F and discussed on page 13 lines 17 to 21 and on page 19, lines 22 to page 21 line 2:

(page 13, lines 17-21)

"In order to investigate whether these findings held true in vivo we investigated if A2MG regulated early neutrophil recruitment to the site of infection during on-going CLP. Administration of A2MG MV prior to CLP led to a dose-dependent increase in the number of peritoneal exudate neutrophils collected at 6 h when compared to mice treated with MV alone (Figure 7F)."

(page 19, lines 22 to page 21 line 2)

"These findings led us to hypothesize that microparticle-A2MG enhanced survival by promoting efficient neutrophil recruitment to the site of infection; a concept that has been postulated for other agents that are protective in sepsis including IL-33 (Alves-Filho et al, 2010). This hypothesis that was corroborated by the in vivo findings with A2MG MV, where administration of these vesicles led to an enhanced and dose dependent increase in neutrophil recruitment to the peritoneum following CLP (Figure 7F)."

4c).What is the effect of an antibody to LRP1 in the adhesion assays?

We have conducted additional experiments to address it. These results are now displayed in Figure 6D and discussed on page 12 second paragraph that reads:

(page 12, 2nd paragraph)

"We next investigated the role of neutrophil LRP1 in mediating the pro-adhesive actions of A2MG MP. Here incubation of neutrophils with an anti-LRP1 antibody led to a significant reduction in

© EMBO

neutrophil adhesion when compared with A2MG MP incubated HUVEC, an effect that was lost when neutrophils were instead incubated with a relevant isotype control (Figure 6D). Together these results indicate that A2MG is transferred by micro particles on to the endothelial plasma membrane where it engages LRP1 on the flowing neutrophils promoting firm leukocyte adhesion."

Referee #2 (Remarks):

In this paper, the authors describe that the acute phase reactant alph-2-macroglobulin (A2MG) has therapeutic effects in sepsis, especially the A2MG that is found bound to micro particles. The data that are presented are of value, but they are too premature to lead to solid conclusions. The main flaws of the paper are the following:

1). In the in vivo experiments (CLP model in mice), a number of essential controls are lacking. It is important that empty micro particles are involved in the study, as well as micro particles uploaded with other proteins than A2MG. Also a comparison with sA2MG can only be made if the correct amount of protein is known that is delivered with the A2MG-E MP.

We agree with the reviewer that empty micro particles are an important control and have conducted additional experiments employing either empty micro vesicles or micro vesicles loaded with A2MG as well as investigated the role of LRP1, the A2M receptor, in mediating the actions of A2MG-E MP in vivo. The vesicles allowed us to directly investigate the actions of A2MG incorporated in micro vesicles in regulating the host responses during bacterial sepsis. These results are now presented in Figure 3, Figure 4B and Supporting Information Figure 5 and described on page 9 in the results subheading 'A2MG micro vesicles display protective action in murine sepsis' that reads:

"Since A2MG micro particles contain a number of other proteins (Dalli et al, 2013) that may also exert protective actions we next constructed synthetic micro vesicles that were selectively loaded with a defined amount of A2MG (A2MG-MV; see methods for details) and tested them in murine sepsis. Administration of A2MG-MV to CLP mice led to a dose dependent reduction in peritoneal exudate neutrophil counts at 12h when compared with mice treated with control micro vesicles (cMV; Figure 3A). Treatment of mice with A2MG-MV also led to a dose-dependent increase in the number of phagocytosed bacteria in murine peritoneal exudates, as determined by in situ hybridization and flow cytometery (Figure 3B). Furthermore, there was a dose dependent reduction in both exudate (Figure 3C) and plasma (Figure 3D) bacterial loads when compared to mice treated with cMV. Administration of A2MG-MV also led to a dose dependent reduction in exudate and plasma levels of pro-inflammatory cytokines, including IL6 and TNF-α (Supporting Information Figure 5) as well as second organ injury, as determined by a reduction in lung MPO levels (Figure 3E). Treatment of mice subjected to CLP with A2MG-MV also significantly protected against hypothermia (Figure 3F). In all cases, doses of 1x105 and 1x106 A2MG-MV elicited the most significant effects. Administration of A2MG-MV (1x106) led to a significant reduction in mortality when compared with mice treated with cMV (Figure 4A). Together these results indicate that A2MG exerts protective actions in sepsis likely mediating at least some of the protective actions displayed by A2MG-E MP (Figures 1 and 2).

To corroborate this further, we next investigated whether the A2MG receptor, the low-density lipoprotein receptor-related protein (LRP) 1, mediated these protective actions. Here we employed an established in vivo transfection system using siRNA to silence LRP1 expression in mice. Mice received either a mock lentiviral vector or a lentiviral vector containing an siRNA sequence to LRP1. Assessment of peripheral blood LRP1 levels 48h after transfection demonstrated a significant reduction in peripheral blood LRP1 expression (~ 50%; P<0.05; Supplementary Information Figure 6), confirming the efficacy of this approach at reducing LRP1 expression in peripheral blood leukocytes. We next subjected mock transfected or siRNA transfected mice to CLP, then treated these mice with either PBS or A2M-E MP and assessed survival over 4 days. There were no significant differences between the two vehicle treated groups with 100% lethality within 52 h (Figure 4). Administration of A2M-E MP to mice treated with mock vector significantly

© EMBO

improved survival, an action that was lost in mice that were also treated with LRP1 siRNA (Figure 4). Together these results indicate that in mice LRP1 mediates the protective actions of A2MG-E MP."

2). In most of the experiments, just one dose of A2MG is used. It is important, in vivo and in vitro, to study dose-response effects in order to be able to draw conclusions.

We have now conducted additional experiments to address this point. The results are displayed in Figure 3, 5, 6 and 7 as well as Supporting Information Figure 5,8,9 and 10 that demonstrate a dose dependent response in protection from sepsis in vivo (using A2MG-MV) as well as bacterial uptake and killing by mouse and human leukocytes (using sA2MG and A2MG micro particles).

3). The basic observations in the mouse CLP model raise interest with the reader, but most of the mechanistic investigations are then done in human cells. All of the in vitro studies should also be performed in mice, for obvious reasons.

We have now conducted additional experiments with mouse macrophages and neutrophils to address this point. The results for these experiments demonstrate that human A2MG also regulates mouse neutrophil and macrophage responses. These results are presented in Supporting Information Figure 9 and Figure 14 and discussed on page 10 line 18-22 and page 13, line 2-5:

(page 10, line 18-22)

"In order to assess the role of A2MG in regulating the leukocyte responses elicited by A2MG MP we next investigated whether sA2MG regulated macrophage and peripheral blood neutrophil phagocytosis and ROS production. Incubation of human and mouse macrophages with sA2MG led to a dose dependent increase in macrophage phagocytosis (Supporting Information Figure 8 and 9).

(page 13, line 2-5)

"The increased CD11b activation was functionally reflected in an enhanced human and mouse neutrophil interaction with ICAM-1, one of the CD11b ligands (Figure 7B, Supporting Information Figure 14).

4). Many of the data displayed in the figures are hard to understand because they are not properly explained in the paper (results, materials or legends). For example, Fig 1A and 1C.

We thank the reviewer for pointing this out and have now endeavoured to improve the clarity of the manuscript by providing additional experimental details as well as expanding on the rationale behind the experiments presented and discussed.

5). Some of the effects of A2MG are blocked by inhibiting the receptor of A2MG, namely the LRP. It is important that as many as possible effects of A2MG are investigated in conditions of LRP inhibition, knockout or knockdown.

We thank the reviewer for raising this important point. In order to address this point we have silenced LRP1 in vivo using a siRNA approach and in vivo transfection that gave a significant attenuation of LRP1 expression in leukocytes. The results of these experiments are displayed in Figures 4B, 5B-E, 6E and Supporting Information Figures 6, 8 and 10 and discussed on page 9 lines 2-16, page 11 line 7 to page 12 line 3 and page 12 second paragraph as follows:

(page 9, lines 2-16)

"To corroborate this further, we next investigated whether the A2MG receptor, the low-density lipoprotein receptor-related protein (LRP) 1, mediated these protective actions. Here we employed an established in vivo transfection system using siRNA to silence LRP1 expression in mice. Mice received either a mock lentiviral vector or a lentiviral vector containing an siRNA sequence to LRP1. Assessment of peripheral blood LRP1 levels 48h after transfection demonstrated a significant reduction in peripheral blood LRP1 expression (~ 50%; $P<0.05$; Supplementary Information Figure 6), confirming the efficacy of this approach at reducing LRP1 expression in peripheral blood leukocytes. We next subjected mock transfected or siRNA transfected mice to CLP, then treated these mice with either PBS or A2M-E MP and assessed survival over 4 days. There were no significant differences between the two vehicle treated groups with 100% lethality within 52 h (Figure 4). Administration of A2M-E MP to mice treated with mock vector significantly improved survival, an action that was lost in mice that were also treated with LRP1 siRNA (Figure 4). Together these results indicate that in mice LRP1 mediates the protective actions of A2MG-E MP."

(page 11 line 7 to page 12 line 3)

"Next we investigated the role of LRP1 in mediating the protective responses of microparticle-A2MG on human primary leukocytes. Incubation of neutrophils with micro particles containing elevated A2MG levels, (termed A2MG MP; see methods and (Dalli et al, 2013) for details) led to a dose dependent increase in bacterial phagocytosis (Figure 5B), an action that was inhibited when leukocytes were pre-incubated with an anti-LRP1 antibody. When human neutrophils were incubated with A2MG MP we found a significant increase in ROS production when the neutrophils were also incubated with E. coli (Figure 5C), an action inhibited when cells were pre-incubated with an anti-LRP1 antibody. Similar results were obtained with macrophages both in terms of bacterial phagocytosis (Figure 5D) and ROS production (Figure 5E). These results indicate that leukocyte LRP1 mediates the protective actions of A2M MP.

In order to assess the role of A2MG in regulating the leukocyte responses elicited by A2MG MP we next investigated whether sA2MG regulated macrophage and peripheral blood neutrophil phagocytosis and ROS production. Incubation of human and mouse macrophages with sA2MG led to a dose dependent increase in macrophage phagocytosis (Supporting Information Figure 8 and 9). Incubation of human macrophages with sA2MG also led to a dose dependent increase in ROS production when the cells were co-incubated with *E. coli*. (Supporting Information Figure 8). These actions of sA2MG on human macrophages were abrogated when the cells were pre-incubated with anti-LRP1 antibody (Supporting Information Figure 8). sA2MG also elicited an increase in bacterial phagocytosis and ROS production with human peripheral blood neutrophils actions that were sensitive to cell incubation with anti-LRP1 antibody (Supporting Information Figure 10)."

(page 12, 2nd paragraph)

"We next investigated the role of neutrophil LRP1 in mediating the pro-adhesive actions of A2MG MP. Here incubation of neutrophils with an anti-LRP1 antibody led to a significant reduction in neutrophil adhesion when compared with A2MG MP incubated HUVEC, an effect that was lost when neutrophils were instead incubated with a relevant isotype control (Figure 6D). Together these results indicate that A2MG is transferred by micro particles on to the endothelial plasma membrane where it engages LRP1 on the flowing neutrophils promoting firm leukocyte adhesion."

6). Since there appear to be differences in effects between A2MG-E micro particles and free sA2MG, it is confusing why in certain experiments only sA2MG is used. There should be better motivation for this choice.

Thank you for raising this point. We added a detailed discussion of this point in the revised manuscript as well as conducted additional experiments to help clarify this point. We have no doubts, and indeed there are scant data in the literature, that A2MG exerts some degree of protection in experimental sepsis. Our new findings with micro particles indicate an enhancement of this effect with the involvement of novel biological actions, perhaps due to better pharmacokinetics. It is true

that micro particles contain many other bioactive molecules and to address this point we have employed novel synthetic micro vesicles that were loaded with A2MG to directly investigate the actions of this novel pathway comparing the responses elicited by these vesicles with empty vesicles. These experiments demonstrate that micro vesicle incorporated A2MG exerts potent protective actions as observed with A2MG-E micro particles. The results of these experiments are displayed in figure 5 and figure 7 as well as Supporting Information Figure 8, 10 and 14 and discussed on page 11 last paragraph to page 12 line 2, page 12 last paragraph to page 13 line 5 and page 18 second paragraph of the revised manuscript as follows:

(page 10, last paragraph to page 12 line 3)

"In order to assess the role of A2MG in regulating the leukocyte responses elicited by A2MG MP we next investigated whether sA2MG regulated macrophage and peripheral blood neutrophil phagocytosis and ROS production. Incubation of human and mouse macrophages with sA2MG led to a dose dependent increase in macrophage phagocytosis (Supporting Information Figure 8 and 9). Incubation of human macrophages with sA2MG also led to a dose dependent increase in ROS production when the cells were co-incubated with E. coli. (Supporting Information Figure 8). These actions of sA2MG on human macrophages were abrogated when the cells were pre-incubated with anti-LRP1 antibody (Supporting Information Figure 8). sA2MG also elicited an increase in bacterial phagocytosis and ROS production with human peripheral blood neutrophils actions that were sensitive to cell incubation with anti-LRP1 antibody (Supporting Information Figure 10)."

(page 12, last paragraph to page 13 line 5)

"We further explored the molecular mechanisms by which A2MG regulated neutrophil recruitment to endothelial cells. For this purpose we incubated human neutrophils with sA2MG, eliminating the possibility that other micro particle derived proteins could confound analyses, and assessed the expression of the active conformation of CD11b, the alpha moiety of Mac-1 that is involved in neutrophil recruitment onto the vascular endothelium (Edens & Parkos, 2003). Neutrophil incubation with sA2MG led to a significant increase in CD11b neo-epitope exposure (Figure 7A). The increased CD11b activation was functionally reflected in an enhanced human and mouse neutrophil interaction with ICAM-1, one of the CD11b ligands (Figure 7B, Supporting Information Figure 14).

(page 18, 2nd paragraph)

"It is noteworthy that sA2MG also displayed mild protective actions in vivo although these were to a lesser extent than those displayed by A2M-E MP, especially in terms of survival and regulation of the host responses (Figure 1 and 2). This is in contrast to results obtained when sA2MG was incubated with isolated leukocytes in vitro where the free protein displayed biological actions of equal magnitude to those the micro particle bound A2MG (Figure 5, Supporting Information Figure 8 and 10). One possible explanation for these differences between in vivo and in vitro actions for sA2MG is that this protein, in its receptor binding conformation as employed in the current experiments, has a very short systemic half-life in the circulation, estimated to be of ~4 min in mice (Imber & Pizzo, 1981) and is primarily cleared by the liver (Shibata et al, 2003). Therefore these results underscore the notion that incorporation of this protein into micro particles maximizes the protective actions exerted by A2MG. This notion was further corroborated by the finding that administration of synthetic micro vesicles containing A2MG also displayed protective actions in murine sepsis and reduced local and systemic inflammation, as well as bacterial burdens in a dose-dependent manner, ultimately leading to enhanced survival (Figure 3 and 4)."

7) More background on A2MG should be proved in the paper.

We agree and have now elaborated on these actions of A2MG in the discussion of the revised manuscript on page 15, second paragraph, to page 16 line 9:

"A2MG is an evolutionarily conserved tetrameric glycoprotein (Qin et al, 2010), expressed by a number of cell types in its native form and is one of the major circulating anti-proteinases in vertebrates (Rehman et al, 2013). Of note A2MG is the only plasma proteinase able to inhibit a wide range of mammalian proteases in addition to parasite-derived proteinases, including those from Trichophyton mentagrophytes (causal agent of ringworm) and the neutral proteinase of Fusiformis nodosus (causal agent of ovine foot-root; for a detailed review (Rehman et al, 2013)). In mammals, A2MG is predominantly found as a heterotetramer of 160-185 kDa subunits. Upon exposure to proteases or reactive amines A2MG changes conformation, thereby initiating high affinity binding to several cytokines including TNF−α and IL-6. This conformational change also exposes a receptor-binding domain on the protein that leads to its rapid clearance via endocytosis (Imber & Pizzo, 1981). This mechanism has been proposed to mediate the protective actions of A2MG in experimental models of sepsis, by quenching circulating cytokine levels (Webb & Gonias, 1998), in addition to the ability of A2MG to sequester proteinases thereby regulating proteinase activity during inflammatory events (reviewed in (Rehman et al, 2013)).

In other systems A2MG exerts protective actions by inhibiting H2O2 production by polymorphonuclear leukocytes, Trypanozoma cruzi induced macrophage and cardiomyocyte apoptosis and clearing unfolded or misfolded proteins from extracellular spaces (reviewed in detail in (Rehman et al, 2013)). Hence, our present findings with A2MG containing micro vesicles and in a clinically relevant model of sepsis suggest a novel mechanism of action for A2MG, whereby incorporation of A2MG in micro particles potentiates the endogenous host responses resulting in enhanced protective actions in sepsis when compared to sA2MG."

2nd Editorial Decision 26 September 2013

Thank you for the submission of your revised manuscript to EMBO Molecular Medicine. We have now received the enclosed reports from two referees who saw your previous submission. As you will see the reviewers are now supportive and I am pleased to inform you that we will be able to accept your manuscript pending final editorial amendments.

Please submit your revised manuscript within two weeks. I look forward to seeing a revised form of your manuscript as soon as possible.

I look forward to reading a new revised version of your manuscript as soon as possible.

***** Reviewer's comments *****

Referee #1 (Remarks):

This manuscript has improved radically and now the paper, to my opinion, is a very nice story that fits perfectly in the scope of the journal.

Referee #2 (Remarks):

I have no additional questions.
