# Supplementary material for: Microparticle alpha-2-macroglobulin enhances pro-resolving responses and promotes survival in sepsis
Source: EMBO Mol Med. 2013 Dec 16;6(1):27–42. doi: 10.1002/emmm.201303503 (PMC3936490; doi:10.1002/emmm.201303503)
Supplement: Supplementary file 2 [file emmm0006-0027-sd2.pdf]

Microparticle Alpha-2-Macroglobulin Enhances Pro-resolving Responses and Promotes Survival in Sepsis.

Jesmond Dalli[1*], Lucy V Norling[1], Trinidad Montero-Melendez[1], Donata Federici Canova[1], Hazem Lashin[1], Anton M Pavlov[2], Gleb B Sukhorukov[2], Charles J Hinds[1,3] and Mauro Perretti[1]

[1]The William Harvey Research Institute, Barts and The London School of Medicine, Queen Mary University of London, Charterhouse Square, London EC1M 6BQ, United Kingdom;
[2] School of Engineering & Materials Science, Queen Mary, University of London, Mile End Road, London E1 4NS

[3]Intensive Care Unit, St. Bartholomew's Hospital, Barts Health, London, United Kingdom.

* Present address: Center for Experimental Therapeutics and Reperfusion Injury, Harvard Institute of Medicine, Brigham and Women's Hospital and Harvard Medical School, Boston, Massachusetts. 02115

Author for correspondence: Mauro Perretti, Centre for Biochemical Pharmacology, The William Harvey Research Institute, Barts and The London School of Medicine, Queen Mary University of London, Charterhouse Square, London EC1M 6BQ, United Kingdom.
Email m.perretti@qmul.ac.uk; Phone no: +44-2078828782; Fax no: +44-207-8826076

Running Title: Protection by microparticle-A2M in sepsis
