# Supplementary material for: Microparticle alpha-2-macroglobulin enhances pro-resolving responses and promotes survival in sepsis
Source: EMBO Mol Med. 2013 Dec 16;6(1):27–42. doi: 10.1002/emmm.201303503 (PMC3936490; doi:10.1002/emmm.201303503)
Supplement: Supplementary file 3 [file emmm0006-0027-sd3.pdf]

Supporting Information Table of Contents

Tables:

Supporting Information Table 1: Healthy volunteers and sepsis patients demographics.
Supporting Information Table 2: No significant difference in the incidence of co-morbidities between the two patient groups within 24h of admission to the intensive care unit.
Supporting Information Table 3: No significant difference in the medications received by the two patient groups prior to blood collection.

Figures

Supporting Information Figure 1. Schematic elucidating microparticle A2MG enrichment.
Supporting Information Figure 2. A2MG-enriched microparticles modulate exudate cytokines levels and stimulate a pro-resolving lipid mediator phenotype.
Supporting Information Figure 3. A2MG does not display direct antibacterial actions.
Supporting Information Figure 4. A2MG-enriched microparticles enhanced monocyte/ macrophage to neutrophil ratio
Supporting Information Figure 5. A2MG microvesicles reduce systemic and local pro-inflammatory cytokine levels
Supporting Information Figure 6. Reduced peripheral blood expression of LRP1 in mice treated with LRP1 shRNA.
Supporting Information Figure 7. LRP1 expression on human and murine neutrophils is regulated by LPS.
Supporting Information Figure 8. sA2MG enhances human macrophage bacterial phagocytosis and ROS production.
Supporting Information Figure 9. sA2MG enhances mouse macrophage bacterial phagocytosis.
Supporting Information Figure 10. sA2MG enhances human neutrophil bacterial phagocytosis and ROS production
Supporting Information Figure 11. sA2MG enhances cathelicidin release by human neutrophils.
Supporting Information Figure 12. A2MG MP do not alter adhesion molecule expression on TNF-α-stimulated HUVEC.
Supporting Information Figure 13. Microparticles-A2MG enhances neutrophil adhesion to activated HUVEC underflow.
Supporting Information Figure 14. A2MG regulates murine leukocyte responses to endotoxin.
