# Supplementary figures and images for: Microparticle alpha-2-macroglobulin enhances pro-resolving responses and promotes survival in sepsis
Source: EMBO Mol Med. 2013 Dec 16;6(1):27–42. doi: 10.1002/emmm.201303503 (PMC3936490; doi:10.1002/emmm.201303503)

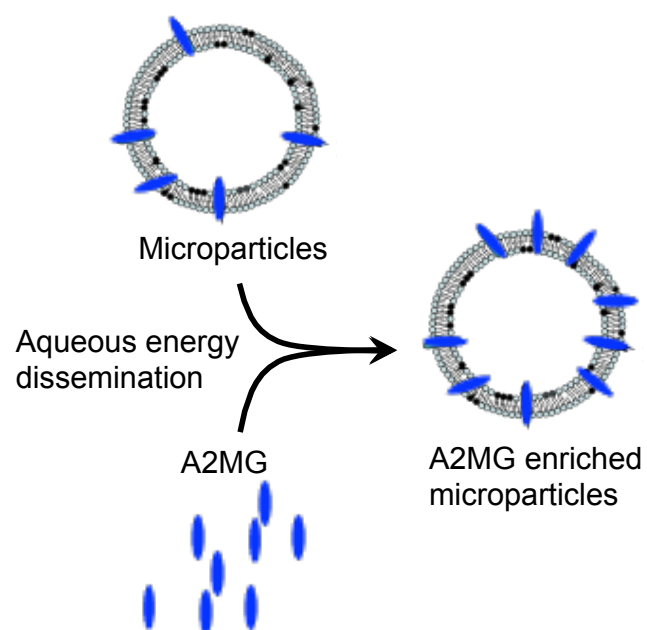

**Supporting Information Figure 1. Schematic elucidating microparticle A2MG enrichment.**

Supplement: Supplementary file 4 [file emmm0006-0027-sd4.pdf]
