# Supplementary material for: Microparticle alpha-2-macroglobulin enhances pro-resolving responses and promotes survival in sepsis
Source: EMBO Mol Med. 2013 Dec 16;6(1):27–42. doi: 10.1002/emmm.201303503 (PMC3936490; doi:10.1002/emmm.201303503)
Supplement: Supplementary file 5 [file emmm0006-0027-sd5.pdf]

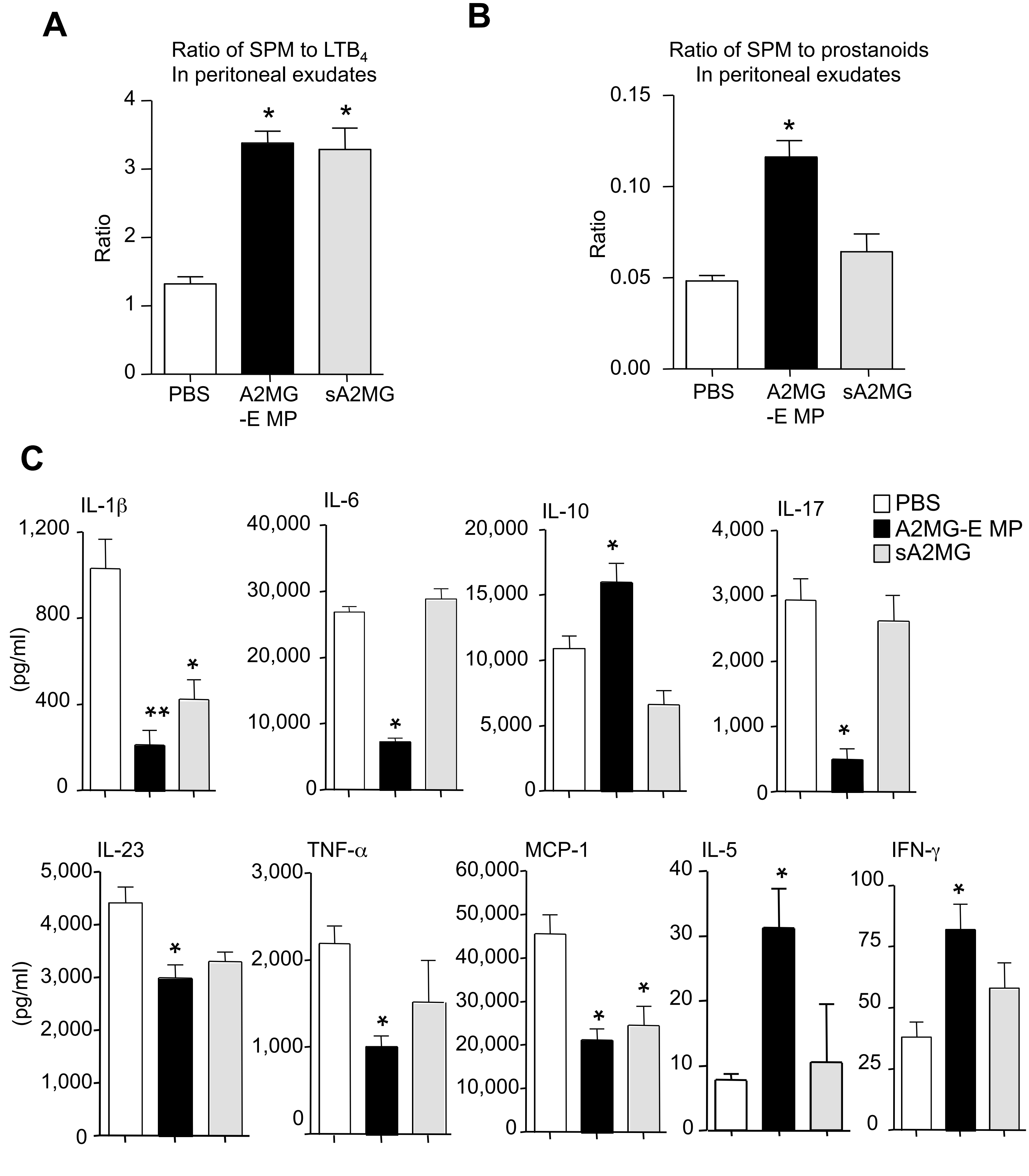

Supporting Information Figure 2. A2MG-enriched microparticles modulate exudate cytokines levels and stimulate a pro-resolving lipid mediator phenotype. Ratio of the total specialized pro-resolving mediators (SPM; RvD1, RvD2, RvD5 and PD1) to (**A**) leukotriene (LT) B_4 and (**B**) prostanoids (PGE_2, PGD_2 and $PGF_{2\alpha}$). (**C**) Exudate cytokine levels determined 12h after CLP by cytokine array. Results are mean ± SEM of 6 mice per group. (*P<0.05 vs. PBS treated mice)**.**
