# Supplementary material for: Microparticle alpha-2-macroglobulin enhances pro-resolving responses and promotes survival in sepsis
Source: EMBO Mol Med. 2013 Dec 16;6(1):27–42. doi: 10.1002/emmm.201303503 (PMC3936490; doi:10.1002/emmm.201303503)
Supplement: Supplementary file 6 [file emmm0006-0027-sd6.pdf]

A

Ampicillin (10µg)	sA2MG (1µg)	sA2MG (100ng)
sA2MG (10ng)	sA2MG (1ng)	Vehicle

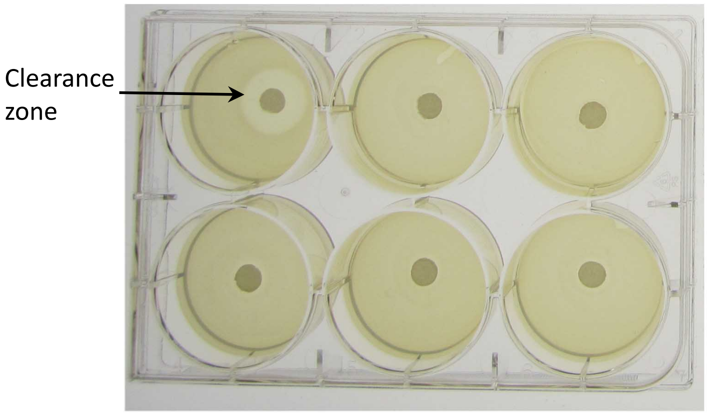

B

Ampicillin (10µg)	sA2MG (1µg)	sA2MG (100ng)
sA2MG (10ng)	sA2MG (1ng)	Vehicle

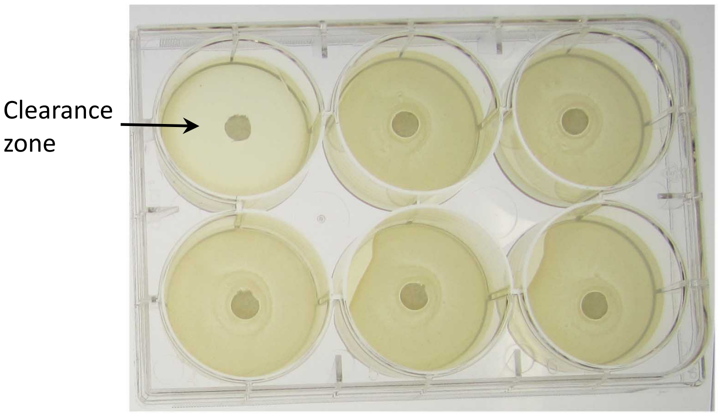

Supporting Information Figure 3. A2MG does not display direct antibacterial actions. 10 µg of ampicillin (as a positive control), sA2MG (1ng-1µg) or vehicle were each placed on LB agar plates containing (**A**) *E. coli* (1.7x10^7 CFU) or (**B**) *S. aureus* (1.7x10^7 CFU)*. The zone of clearance* was assessed after overnight incubation at 37°C. Photographs represent n=3.
