# Supplementary material for: Microparticle alpha-2-macroglobulin enhances pro-resolving responses and promotes survival in sepsis
Source: EMBO Mol Med. 2013 Dec 16;6(1):27–42. doi: 10.1002/emmm.201303503 (PMC3936490; doi:10.1002/emmm.201303503)
Supplement: Supplementary file 7 [file emmm0006-0027-sd7.pdf]

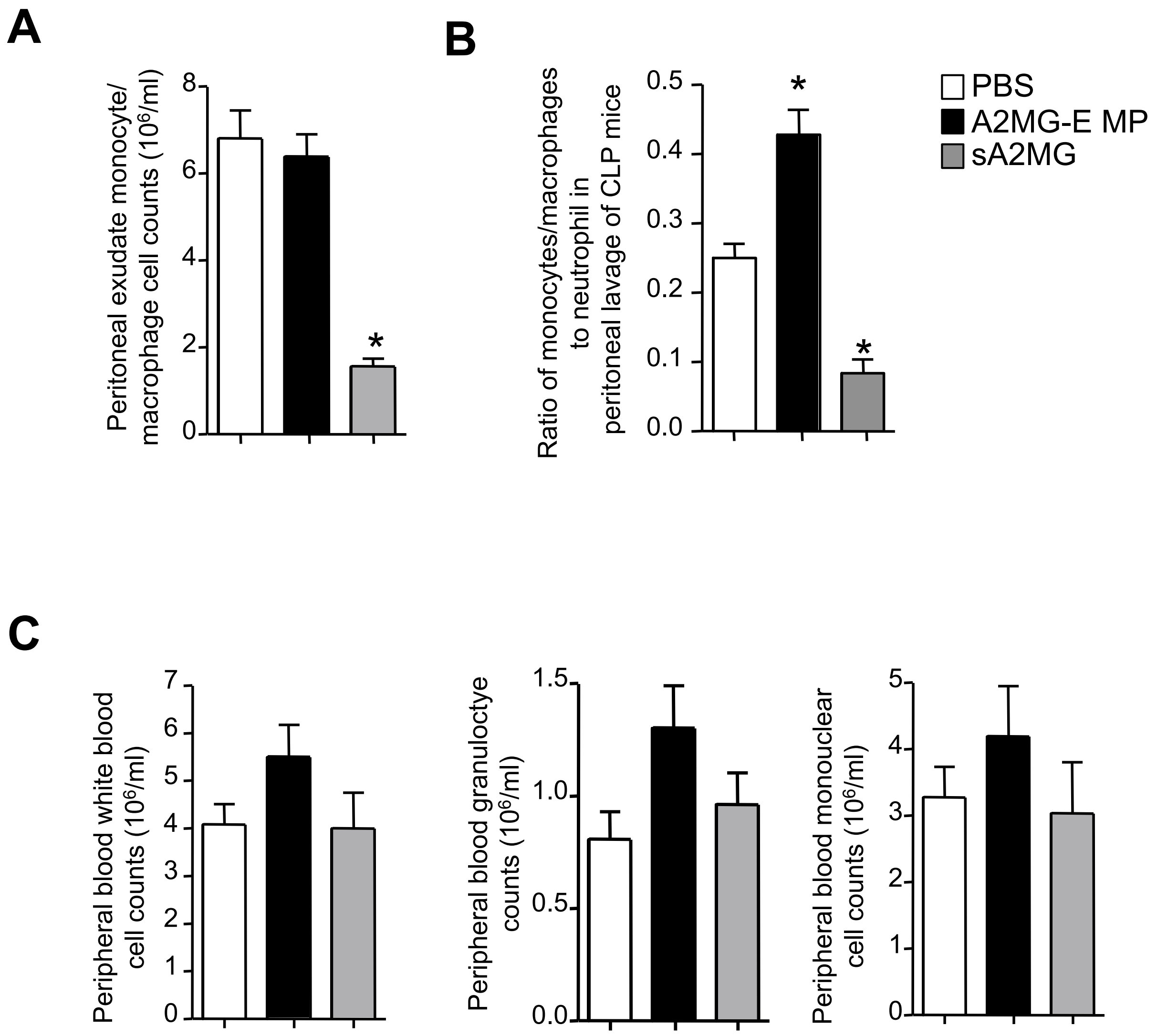

Supporting Information Figure 4. A2MG-enriched microparticles enhanced monocyte/macrophage to neutrophil ratio. CLP mice (see Methods for details) were treated 1h post-surgery with PBS (100µl/mouse i.v.), A2MG-E MP (1x10^5 microparticles/ mouse i.v.) or sA2MG (0.5µg/mouse). (A) The number of monocyte/macrophages at 12h were determined by light microscopy and flow cytometry (see methods for details). (B) Ratio of monocyte/macrophage to neutrophils in peritoneal exudates. (C) Peripheral white blood cell counts at 12h post CLP determined by light microscopy. Results are mean ± SEM of 6 mice per group. (**P*<0.05 vs PBS treated group).
