# Supplementary material for: Microparticle alpha-2-macroglobulin enhances pro-resolving responses and promotes survival in sepsis
Source: EMBO Mol Med. 2013 Dec 16;6(1):27–42. doi: 10.1002/emmm.201303503 (PMC3936490; doi:10.1002/emmm.201303503)
Supplement: Supplementary file 8 [file emmm0006-0027-sd8.pdf]

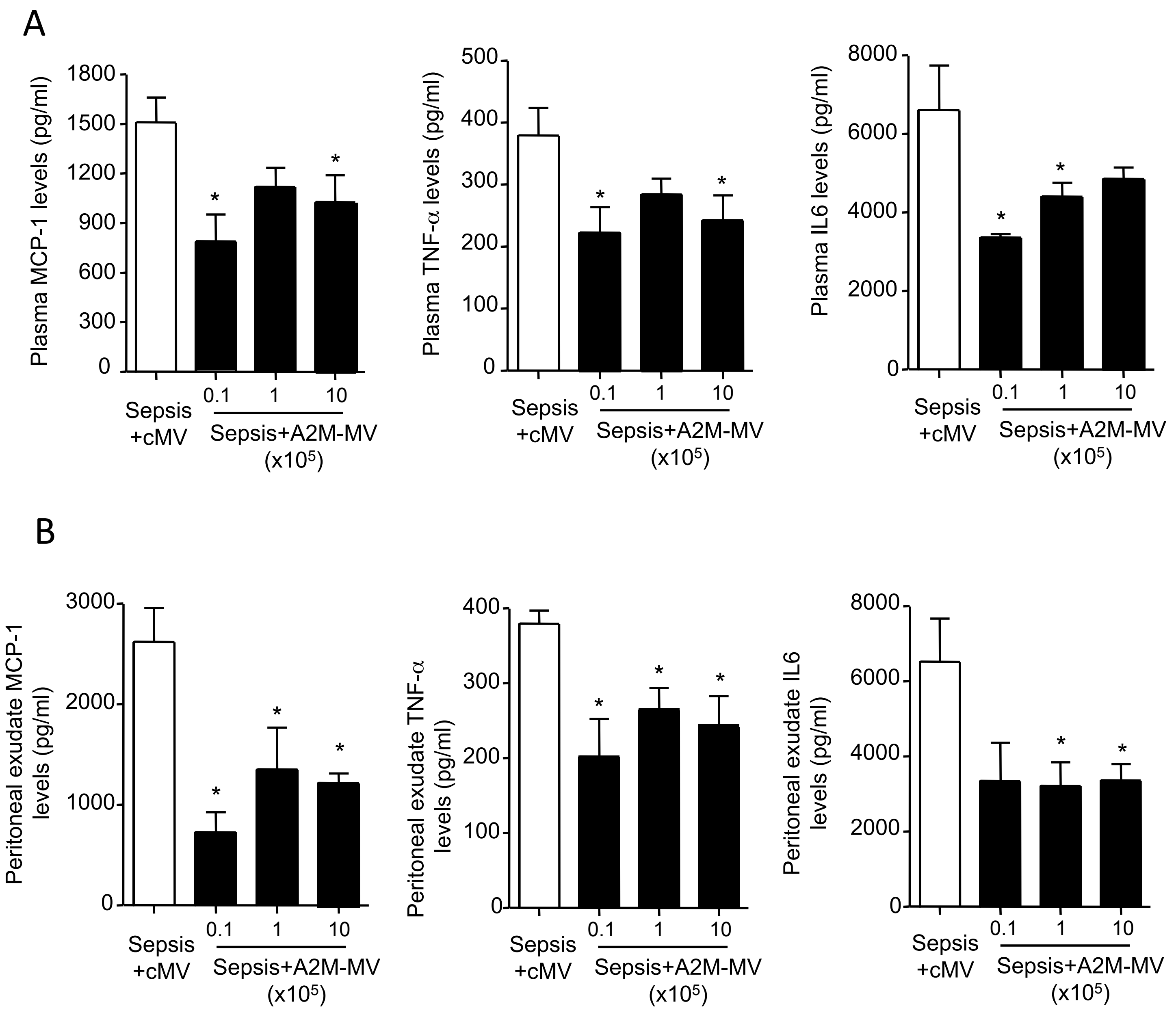

Supporting Information Figure 5. A2MG microvesicles reduce systemic and local pro-inflammatory cytokine levels during sepsis. Controlmicrovesicles (cMV; 10^6 per mouse) or microvesicles containing A2MG (A2MG-MV) were administered *i.v.* at the indicated doses 5min prior to CLP (see Methods for details). Mice were sacrificed at 12h and peritoneal exudate cytokine levels were determined by multiplex ELISA. Results are mean±SEM. n = 5 mice per group (*P<0.05 *vs.* cMV treated mice by one way ANOVA)
