# Supplementary material for: Microparticle alpha-2-macroglobulin enhances pro-resolving responses and promotes survival in sepsis
Source: EMBO Mol Med. 2013 Dec 16;6(1):27–42. doi: 10.1002/emmm.201303503 (PMC3936490; doi:10.1002/emmm.201303503)
Supplement: Supplementary file 9 [file emmm0006-0027-sd9.pdf]

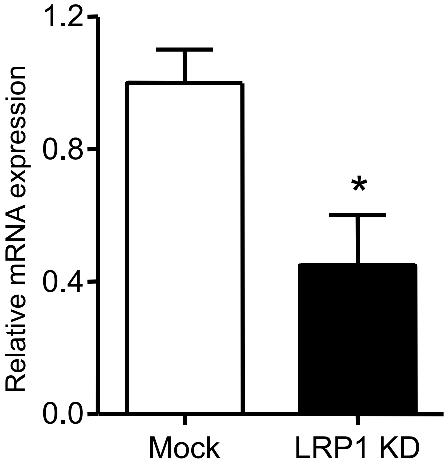

Supporting Information Figure 6. Reduced peripheral blood expression of LRP1 in mice treated with LRP1 shRNA. FvB male mice were transfected with mock or mouse LRP1 shRNA (see methods for details). Blood was collected after 48h, RNA was extracted and LRP1 mRNA levels were assessed by realtime PCR. Results are mean±SEM. n=4-5 mice per group. (**P*<0.05 *vs* Mock transfected group).
