# Supplementary material for: Microparticle alpha-2-macroglobulin enhances pro-resolving responses and promotes survival in sepsis
Source: EMBO Mol Med. 2013 Dec 16;6(1):27–42. doi: 10.1002/emmm.201303503 (PMC3936490; doi:10.1002/emmm.201303503)
Supplement: Supplementary file 10 [file emmm0006-0027-sd10.pdf]

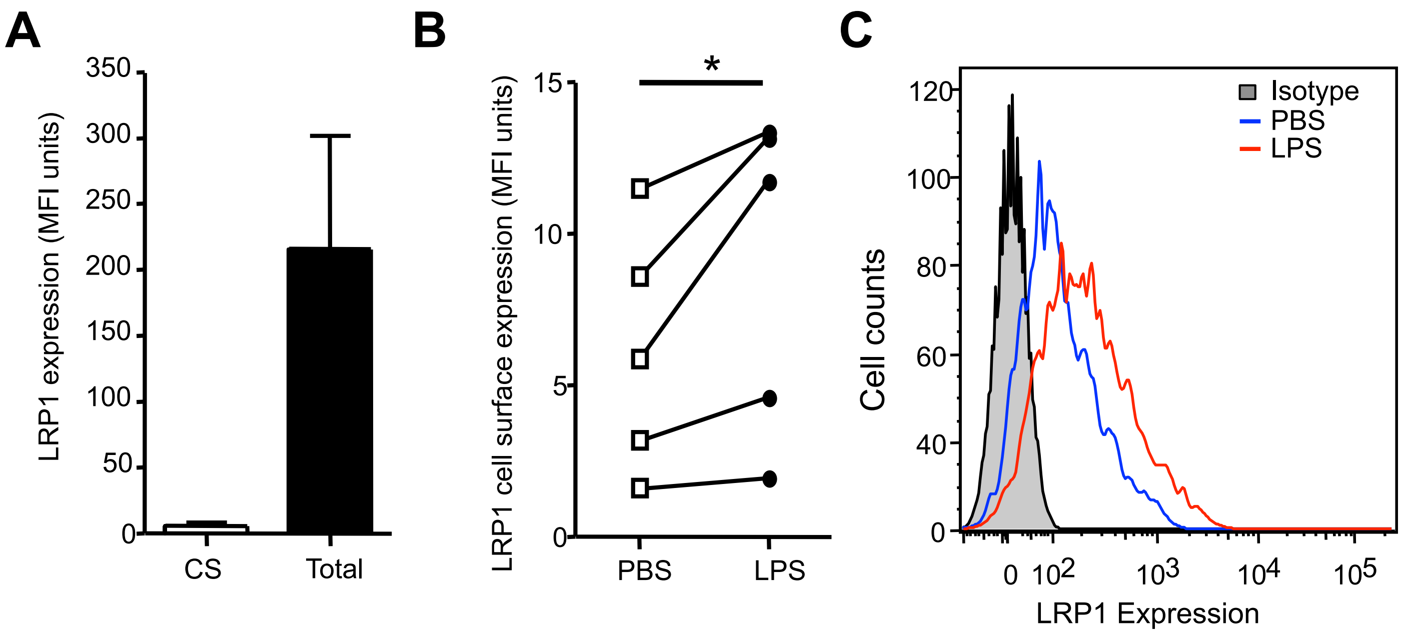

Supporting Information Figure 7. LRP1 expression on human and murine neutrophils is regulated by LPS. (A) LRP1 cell surface (CS) and Total (Fixed and permeabilized cell) expression in resting peripheral blood neutrophils. (B) LRP1 cell surface expression on PBS and LPS (1µg/ml, 30min 37°C) treated human peripheral blood neutrophils. Results are mean ± SEM. . n = 5 distinct cell preparations. (*P<0.05 vs PBS incubated neutrophils by paired student t-Test). (C) Flow-cytometric assessment of murine peripheral blood neutrophils for LRP1 expression with or without LPS (1µg/ml, 60min, 37°C). Results are representative of 3 distinct mouse neutrophil preparations.
