# Supplementary material for: Microparticle alpha-2-macroglobulin enhances pro-resolving responses and promotes survival in sepsis
Source: EMBO Mol Med. 2013 Dec 16;6(1):27–42. doi: 10.1002/emmm.201303503 (PMC3936490; doi:10.1002/emmm.201303503)
Supplement: Supplementary file 11 [file emmm0006-0027-sd11.pdf]

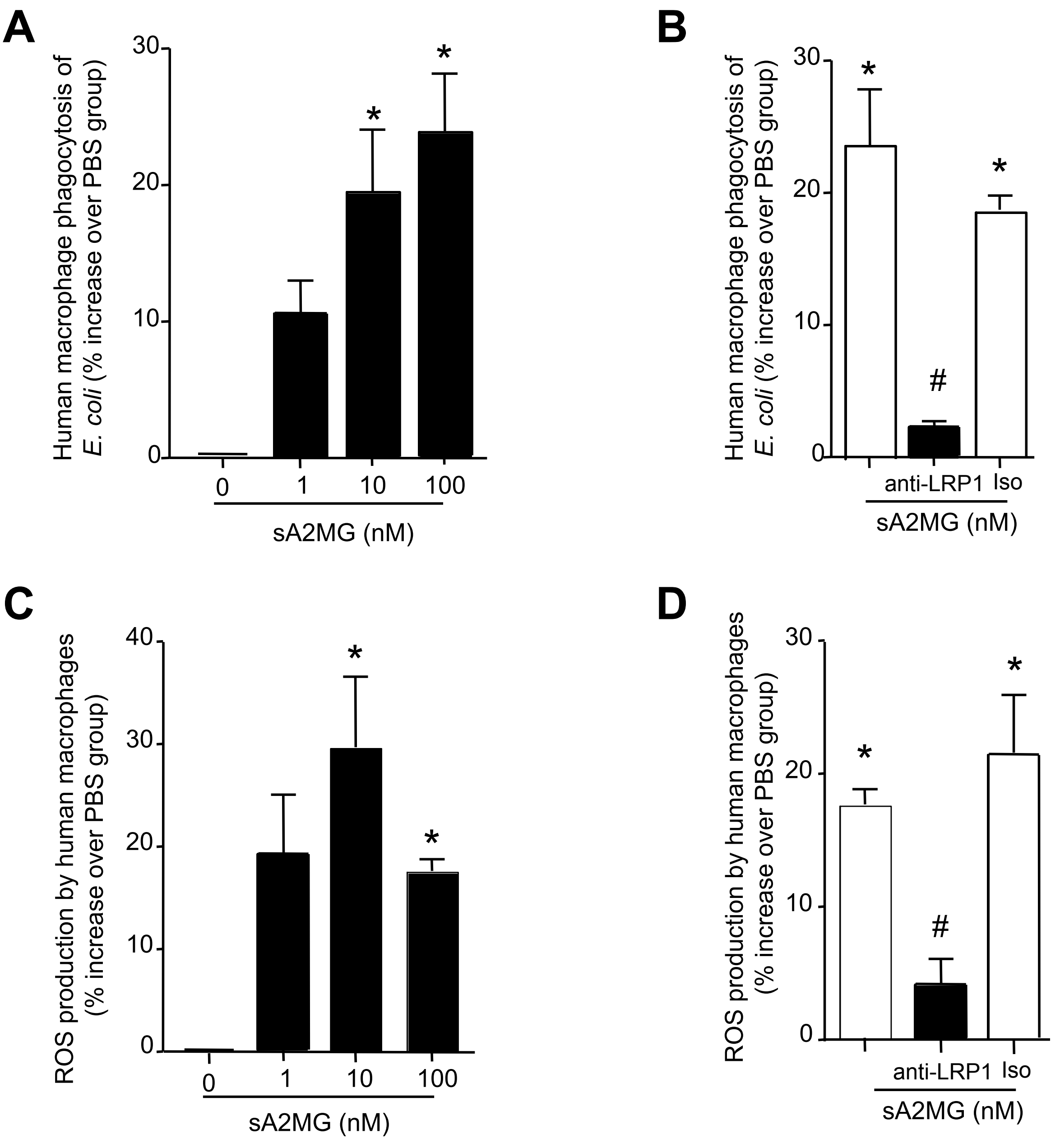

Supporting Information Figure 8. sA2MG enhances human macrophage bacterial phagocytosis and ROS production. (A) Primary human macrophages (1x10^4/well) were plated in 96 well plates and incubated with PBS or sA2MG (1-100nM) 15 min prior to the addition of BacLight labeled E.coli (5x10^5/well) for 60 min (37°C). In some instances anti-LRP1 (1μg/ml) antibody was added 30min prior to the addition of A2MG (right panel). (C) Primary human macrophages (1x10^4/well) were incubated CM-H$_2$DCFDA for 30 min prior to plating in 96 well plates. These were then incubated with PBS or sA2MG (1-100nM) 15 min prior to the addition of *E.coli* (5x10^5/well) for 30 min (37°C) and intracellular ROS production was determined on a plate reader. In some instances anti-LRP1 (1μg/ml) antibody was added 30min prior to the addition of sA2MG (right panel).Results are mean ± SEM of 3 individual cell preparations (*P<0.05, **P<0.01 *vs.* PBS incubation; $^{\#}$ P<0.05 vs sA2MG incubation by one way ANOVA)
