# Supplementary material for: Microparticle alpha-2-macroglobulin enhances pro-resolving responses and promotes survival in sepsis
Source: EMBO Mol Med. 2013 Dec 16;6(1):27–42. doi: 10.1002/emmm.201303503 (PMC3936490; doi:10.1002/emmm.201303503)
Supplement: Supplementary file 12 [file emmm0006-0027-sd12.pdf]

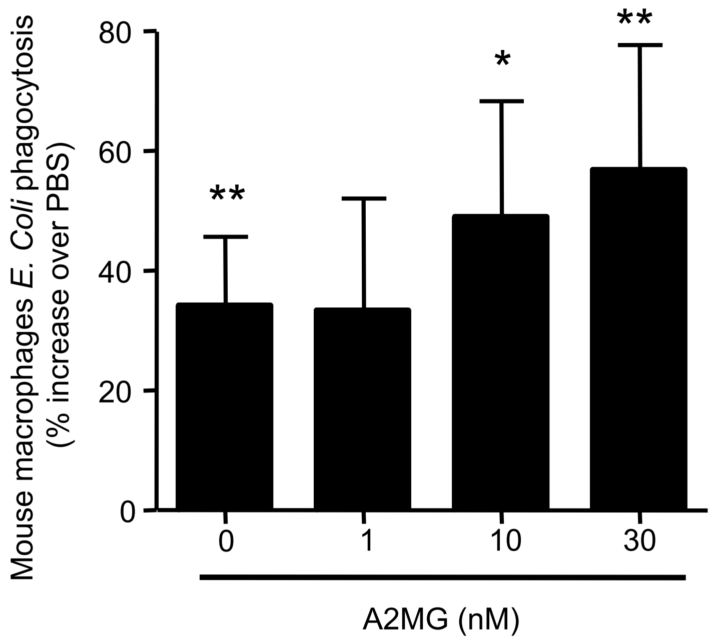

Supporting Information Figure 9. sA2MG enhances mouse macrophage bacterial phagocytosis. Mouse primary macrophages were plated in 96 well plates and incubated with either PBS or A2MG at the indicated concentrations (37°C, 24h) prior to the addition of BacLight labeled E.coli ($5x10^5$/well) for 60 min (37°C). The extent of phagocytosis was determined by measuring total fluorescence (Ex 495/Em535 nm) using a fluorescent plate reader. Results are mean ± SEM of 5 individual cell preparations (*P<0.05, **P<0.01 *vs.* PBS incubation by one way ANOVA)
