# Supplementary material for: Microparticle alpha-2-macroglobulin enhances pro-resolving responses and promotes survival in sepsis
Source: EMBO Mol Med. 2013 Dec 16;6(1):27–42. doi: 10.1002/emmm.201303503 (PMC3936490; doi:10.1002/emmm.201303503)
Supplement: Supplementary file 14 [file emmm0006-0027-sd14.pdf]

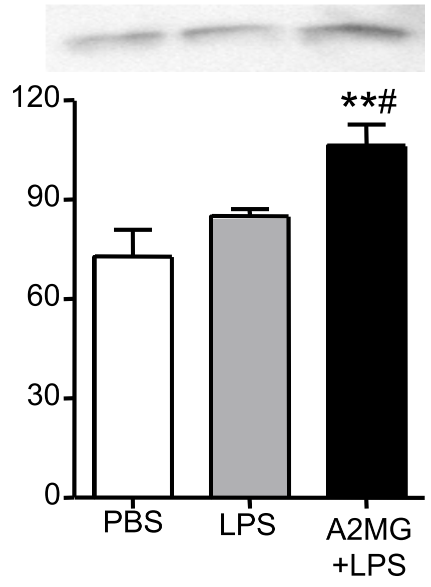

Supporting Information Figure 11. sA2MG enhances cathelicidin release by human neutrophils. Peripheral blood human neutrophils ($3x10^7$/ml) were incubated with cytochalasin B (5min, 37°C pH 7.45), then the cells were incubated for a further 30min with PBS, LPS (1µg/ml) or LPS (1µg/ml) and A2MG (10nM). Cells were then centrifuged, supernatants collected and cathelicidin levels in these supernatants were assessed by western bottling. Inset top is a representative blot. Results are mean ± SEM. n=3 neutrophil preparations. (**P<0.05 vs PBS neutrophils; #P<0.05 vs LPS neutrophils by One Way Anova.)
