# Supplementary material for: Microparticle alpha-2-macroglobulin enhances pro-resolving responses and promotes survival in sepsis
Source: EMBO Mol Med. 2013 Dec 16;6(1):27–42. doi: 10.1002/emmm.201303503 (PMC3936490; doi:10.1002/emmm.201303503)
Supplement: Supplementary file 15 [file emmm0006-0027-sd15.pdf]

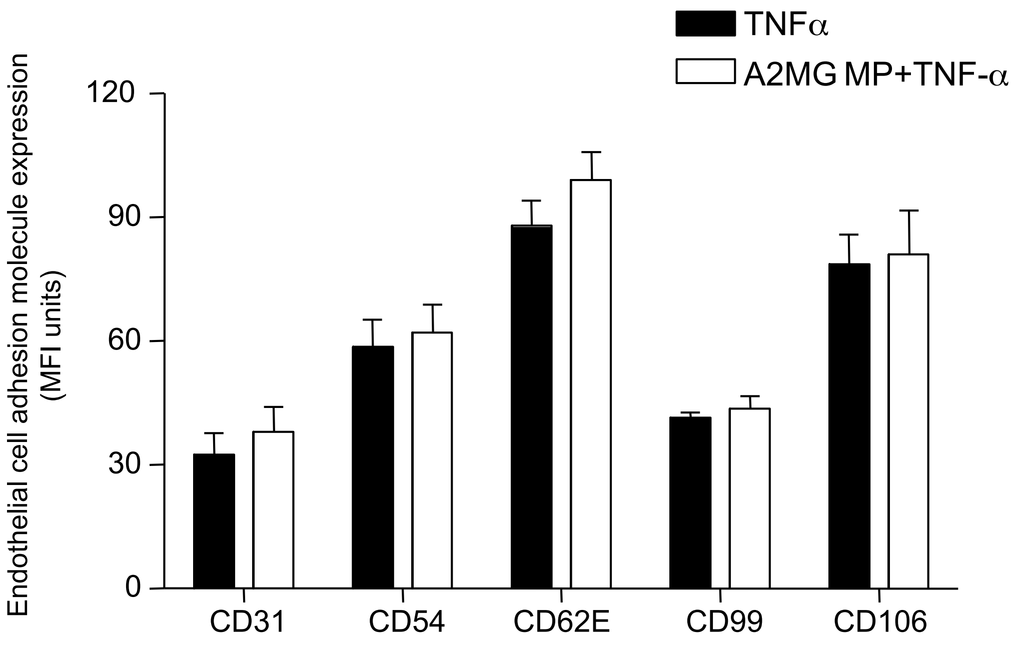

Supporting Information Figure 12. A2MG MP do not alter adhesion molecule expression on TNF-α-stimulated HUVEC. HUVEC were incubated for 4h with buffer, TNF-α (10ng/ml) or TNF-α and A2MG MP ($8x10^5/9.6cm^2$ well). Subsequently, cells were detached and cell-surface expression for the indicated adhesion molecules was assessed after incubation with fluorescently conjugated antibodies by flow cytometry. Results are mean ± SEM. n=3 distinct HUVEC and MP preparations.
