# Supplementary material for: Microparticle alpha-2-macroglobulin enhances pro-resolving responses and promotes survival in sepsis
Source: EMBO Mol Med. 2013 Dec 16;6(1):27–42. doi: 10.1002/emmm.201303503 (PMC3936490; doi:10.1002/emmm.201303503)
Supplement: Supplementary file 16 [file emmm0006-0027-sd16.pdf]

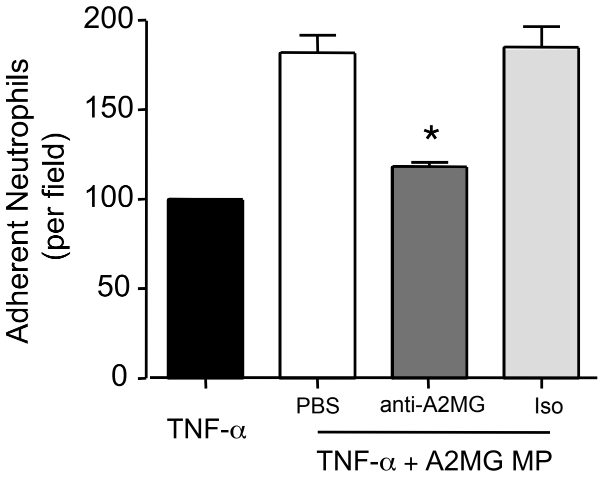

Supporting Information Figure 13. Microparticles-A2MG enhances neutrophil adhesion to activated HUVEC underflow. HUVEC incubated with TNF-α and A2MG MP ($5x10^4/0.6cm^2$) for 4h then incubated for the last 15 min with anti-A2MG antibody or relevant isotype control, prior to perfusion with freshly prepared neutrophils at $1dyne/cm^2$ for 8min, and the number of adherent cells quantified. Results are mean ± SEM. n=6 individual cell and microparticle preparations (*P<0.05 *vs.* Isotype control antibody incubation).
