# Supplementary material for: Microparticle alpha-2-macroglobulin enhances pro-resolving responses and promotes survival in sepsis
Source: EMBO Mol Med. 2013 Dec 16;6(1):27–42. doi: 10.1002/emmm.201303503 (PMC3936490; doi:10.1002/emmm.201303503)
Supplement: Supplementary file 17 [file emmm0006-0027-sd17.pdf]

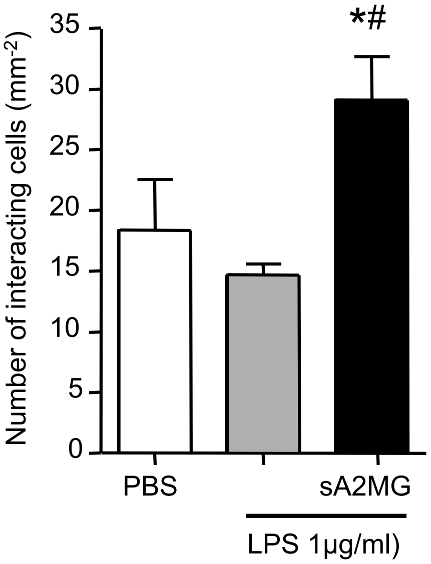

Supporting Information Figure 14. A2MG regulates murine leukocyte responses to endotoxin. Mouse neutrophil interaction with ICAM-1 coated chambers following incubation with LPS (1µg/ml, 60min, 37°C) or A2MG (10nM, 15min, 37°C) followed by LPS (1µg/ml, 60min, 37°C) under flow.
