# Supplementary material for: Microparticle alpha-2-macroglobulin enhances pro-resolving responses and promotes survival in sepsis
Source: EMBO Mol Med. 2013 Dec 16;6(1):27–42. doi: 10.1002/emmm.201303503 (PMC3936490; doi:10.1002/emmm.201303503)
Supplement: Supplementary file 18 [file emmm0006-0027-sd18.pdf]

A

Supporting Information Table 1: Healthy volunteers and sepsis patients demographics.

Sample	PMN counts (10^6 per ml)	Age (years)	Male/ Female	Total microparticle count ($x10^8$/ml)
Healthy Volunteer (HV) Plasma	6.7 ± 1.6	41 ± 4	7/9	14.5 ± 0.7
Sepsis Survivors (SS) Plasma	11.0 ± 1.2	65 ± 4	9/16	16.0 ± 2.0
Sepsis Non-Survivors (SNS) Plasma	13.2 ± 1.1	63 ± 4	16/9	15.5 ± 1.6

[a]Results are mean ± SEM. n=15 for HV and n=25 for sepsis patients.
