# Supplementary material for: Microparticle alpha-2-macroglobulin enhances pro-resolving responses and promotes survival in sepsis
Source: EMBO Mol Med. 2013 Dec 16;6(1):27–42. doi: 10.1002/emmm.201303503 (PMC3936490; doi:10.1002/emmm.201303503)
Supplement: Supplementary file 19 [file emmm0006-0027-sd19.pdf]

Supporting Information Table 2: No significant difference in the incidence of co-morbidities between the two patient groups within 24h of admission to the intensive care unit.

Disease	Sepsis Survivors	Sepsis Non Survivors
Heart/Vascular Disease	9/25	8/25
Respiratory Disease	10/25	11/25
Neurological Disease	0/25	4/25
Gastro Intestinal Disease	3/25	5/25
Cancer	5/25	2/25
Diabetes/ Endocrine Disease	1/25	4/25
