# Supplementary material for: Microparticle alpha-2-macroglobulin enhances pro-resolving responses and promotes survival in sepsis
Source: EMBO Mol Med. 2013 Dec 16;6(1):27–42. doi: 10.1002/emmm.201303503 (PMC3936490; doi:10.1002/emmm.201303503)
Supplement: Supplementary file 20 [file emmm0006-0027-sd20.pdf]

Supporting Information Table 3: No significant difference in the medications received by the two patient groups prior to blood collection.

Treatment	Sepsis Survivors	Sepsis Non Survivors
Activated Protein C	2/25	1/25
Corticosteroids	2/25	3/25
Antibiotics	25/25	25/25
Other	0/25	0/25
